# Artificial Intelligence-Enabled Medical Diagnosis in the Internet of Medical Things: An End-to-End Survey of Connected Sensing, Distributed Intelligence, Clinical Applications, and Validation

**DOI:** 10.3390/diagnostics16193171

**Published:** 2026-09-29

**Authors:** Ahlem Aziz, Seydi Kaçmaz, Azam Isam Abdulkareem Aladwani

**Affiliations:** 1 Electrical and Electronics Engineering Department, Karabük University, 78050 Karabük, Türkiye; 2 Department of Electrical and Electronics Engineering, Faculty of Engineering, Gaziantep University, 27310 Gaziantep, Türkiye; seydikacmaz@gantep.edu.tr; 3 Department of Drone Techniques Engineering, Technical College of Mosul, Northern Technical University, Mosul 41001, Iraq; azzam.esam@ntu.edu.iq

**Keywords:** Internet of Medical Things, medical diagnosis, diagnostic artificial intelligence, clinical decision support, machine learning, deep learning, federated learning, edge intelligence, biosensors, point-of-care testing, external validation

## Abstract

The Internet of Medical Things (IoMT) can extend diagnostic observation beyond episodic encounters by connecting wearable, point-of-care, imaging, and bedside devices to artificial-intelligence services. Yet continuous measurement is not itself diagnosis: a clinically useful system must establish signal validity, map an output to a defined diagnostic role, communicate uncertainty, and demonstrate agreement with an appropriate reference standard. This survey analyzes the complete IoMT diagnostic pathway from acquisition and quality control through edge–fog–cloud computation, machine and deep learning, federated training, and clinician-governed action. It distinguishes monitoring, screening, early detection, differential diagnosis, prognosis, and decision support because these tasks require different labels, thresholds, metrics, and evidence. Evidence is synthesized across electrocardiography and cardiovascular risk, glucose and metabolic assessment, connected imaging and oncology, respiratory and infectious-disease detection, electroencephalography and seizure recognition, and decentralized point-of-care testing. The analysis shows how sensor placement, missingness, compression, latency, privacy mechanisms, and site heterogeneity can change diagnostic performance even when the model is unchanged. A diagnostic evidence framework is therefore proposed that links reference standards and patient-level data separation to sensitivity, specificity, calibration, external validation, target-device feasibility, uncertainty-based referral, and prospective workflow utility. Security, explainability, and lifecycle monitoring are treated as conditions of diagnostic reliability rather than independent features. The resulting synthesis provides a clinically centered basis for deciding which IoMT outputs may support screening or diagnosis, which remain monitoring signals, and what evidence is still required before deployment.

## 1. Introduction

Connected medical devices are changing where diagnostic evidence is acquired. Electrocardiograms, oxygen saturation, glucose trajectories, respiratory sounds, microscopy images, and point-of-care biomarker measurements can now originate at the bedside, in an outpatient clinic, or in a patient’s home. The Internet of Medical Things (IoMT) links these instruments to computation and clinical information systems, potentially increasing temporal coverage and shortening the distance between an abnormal observation and review. Its clinical value, however, depends on whether the connected pathway preserves the meaning of the measurement from the patient to the diagnostic decision; connectivity alone cannot convert a physiological stream into a validated diagnosis [1,2].

This distinction is central to the present survey. A wearable that records a variable is a monitoring device; an algorithm that flags an unusual window performs detection; a screening system identifies people who may require confirmatory testing; and a diagnostic system estimates the presence, subtype, or severity of a condition against a clinical reference standard. Prognostic models answer a different question by estimating a future outcome, whereas clinical decision support can combine any of these outputs with patient context without replacing clinician judgment. Reviews of AI and IoMT describe substantial opportunities across these functions, but frequently combine them under broad terms such as prediction or smart healthcare, making the required evidence difficult to interpret [3,4].

### 1.1. The Data Deluge and the Case for Machine Intelligence

The scale problem is not merely the number of samples. IoMT data are heterogeneous in modality, sampling rate, placement, missingness, and clinical context; a model may therefore learn sensor identity, acquisition site, or care setting instead of disease. Learning-based systems can discover temporal and spatial patterns that fixed thresholds miss, but the inference remains diagnostically meaningful only when signal quality and the reference label are controlled [5]. In medical imaging, narrowly defined AI systems can approach specialist performance under selected evaluation conditions, yet this does not establish diagnostic equivalence across populations or workflows. The relevant innovation is a bounded model whose errors, uncertainty, and influence on downstream decisions are measured [6].

Connected sensing also creates diagnostic opportunities that are not available from a single encounter. Repeated electrocardiogram (ECG) recordings or glucose measurements may reveal intermittent events and trajectories, while decentralized biosensors can bring molecular or infectious-disease testing closer to the patient. The COVID-19 literature illustrates the complementarity: molecular assays, antigen–antibody and CRISPR biosensors address biological detection, whereas AI-based analysis can support interpretation of images or other measurements; these are different evidence streams and should not be presented as interchangeable tests [7]. In leukemia, emerging point-of-care platforms similarly target fusion genes, surface antigens, exosomes, and circulating tumor cells, demonstrating that the IoMT diagnostic layer can include molecular measurements rather than only wearables [8].

### 1.2. Why Intelligence Must Be Distributed

Early deployments sent measurements to the cloud for analysis. This arrangement is simple, but it can be poorly matched to seconds-critical arrhythmia or seizure alerts, intermittent connectivity, and minimization of identifiable raw data. Edge-AI reviews consequently report migration of quality assessment and preliminary inference toward near-patient hardware, with more computationally demanding analysis and longitudinal aggregation retained upstream [9]. The diagnostic implication is not that edge computation is always superior: a local model can reduce delay, but limited memory and aggressive compression may reduce sensitivity or calibration. Compute placement must therefore follow the clinical deadline and the cost of false reassurance, not a general preference for edge or cloud [10].

**Figure 1:**
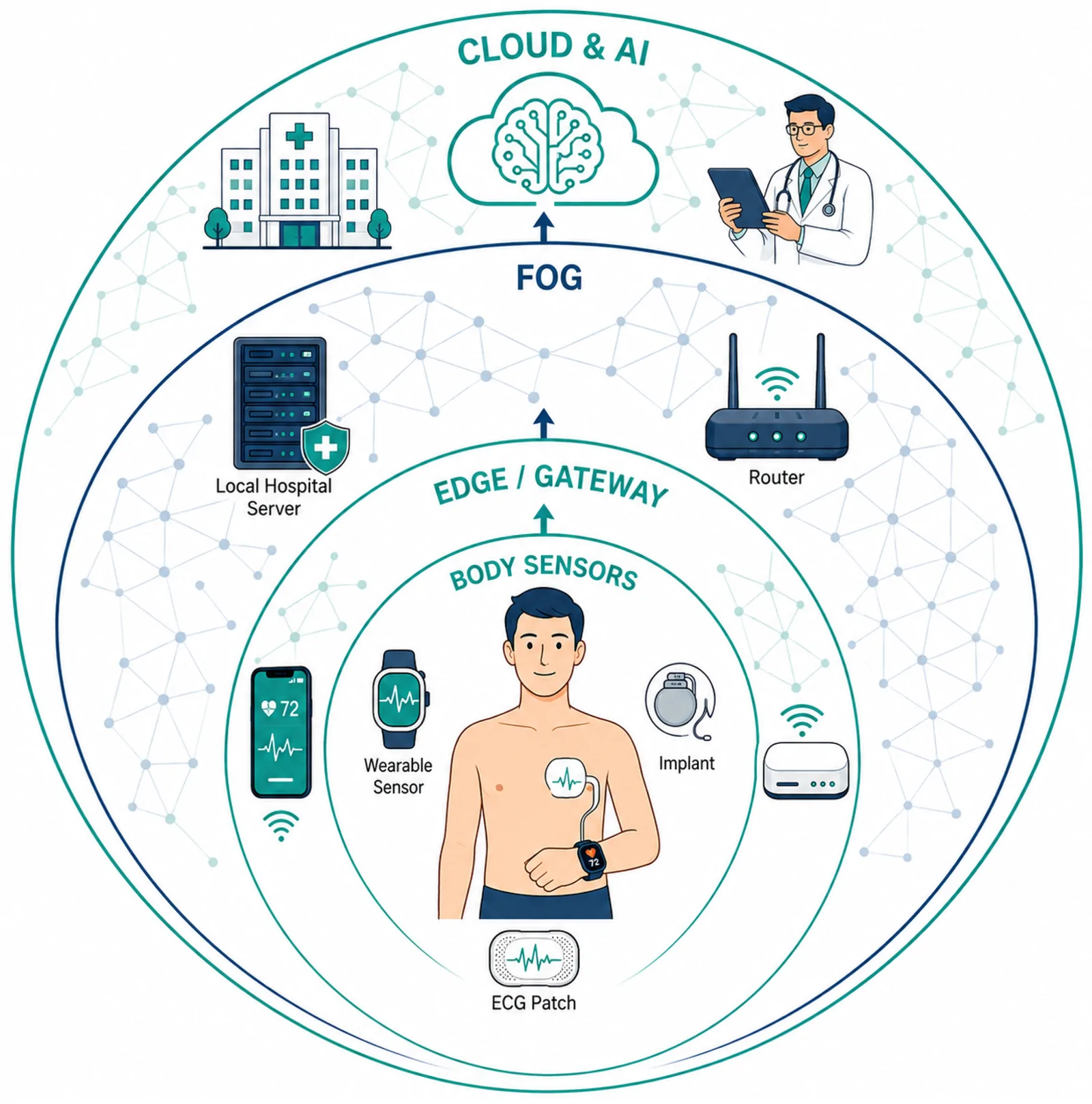
The diagnostic IoMT ecosystem. Physiological, imaging, and molecular measurements move through edge, fog, and cloud services for quality control and analysis; outputs enter screening, diagnostic, prognostic, or decision-support pathways under clinician governance.

Figure 1 summarizes the diagnostic IoMT ecosystem considered in this survey.

Table 1 establishes the terminology used throughout this survey. The categories can occur in one pathway, but they are not interchangeable. For example, continuous ECG monitoring may trigger an arrhythmia screen; a clinician then interprets the trace with symptoms and prior records before establishing a diagnosis. A prognosis may be generated only after diagnosis, and a decision-support system may recommend escalation while explicitly deferring the final decision. Conflating these stages exaggerates clinical claims and obscures which reference standard and error costs apply.

### 1.3. Scope, Contributions, and Relation to Prior Surveys

Existing surveys provide strong but usually sectional views of the field. Federated-learning reviews examine decentralized optimization and privacy [11,12]; security reviews organize attacks and defenses [13,14]; biosensor reviews concentrate on the sensing interface [15]; and application-specific reviews can analyze one domain in depth [16]. Broader surveys connect AI to enabling technologies and clinical domains [3,4,17], but the diagnostic claim often remains implicit: monitoring, anomaly detection, prediction, and diagnosis are compared using model scores without tracing how labels were established or how outputs enter confirmatory care. Table 2 compares the scope coverage of representative surveys across six selected dimensions.

*Research gap and thesis.* Diagnostic performance is a property of a pathway, not of an isolated classifier. Sensor quality determines which biological variation is observable; preprocessing can suppress diagnostically relevant morphology; compute placement changes latency and missingness; privacy and compression change the learned representation; and the workflow determines whether an uncertain output is reviewed, repeated, or ignored. The central thesis of this survey is therefore that an AI-enabled IoMT system should be evaluated from *measurement to reference standard to accountable clinical action*. Three coupled engineering trade-offs remain useful—latency versus accuracy, energy versus privacy, and model size versus robustness to domain shift—but they are interpreted here through their effect on sensitivity, specificity, calibration, and safe referral (Table 3).

*Contributions.* This survey contributes four elements. First, it defines the diagnostic roles that connected AI may legitimately perform and separates them from general monitoring. Second, it follows diagnostic information end to end, linking sensing, preprocessing, architecture, learning, and federation to clinical error rather than discussing these components as independent technologies. Third, it compares evidence across cardiovascular, metabolic, oncological, respiratory, infectious, neurological, and point-of-care pathways, including connected ECG, imaging, EEG, and biomarker analysis. Fourth, it proposes a diagnostic evidence framework that joins patient-level separation, reference standards, calibration, external and temporal validation, uncertainty-based referral, target-device testing, and prospective workflow evaluation. The remainder of the paper concentrates these dimensions on diagnostic translation.

The “Deep” ratings assigned to the present survey in Table 2 were determined with the same scope-coverage rubric applied to the comparison surveys and indicate breadth of treatment rather than superior methodological quality. The sensing/wearables rating is supported by the dedicated analysis of acquisition modalities, signal quality, sampling, artifacts, and device constraints in Section 5. The edge–fog–cloud rating reflects the separate architectural analysis of placement, latency, queueing, resource limits, and split inference in Section 4. The ML/DL rating is based on the treatment of classical and deep models, compression, calibration, data leakage, and transportability in Section 6; the federated-learning rating reflects the dedicated analysis of optimization, statistical heterogeneity, privacy, communication, and clinical validation in Section 7. The clinical-domains rating is supported by the cross-domain synthesis in Section 9, which distinguishes cardiovascular, metabolic, oncological, respiratory, infectious, neurological, and point-of-care pathways. Finally, the trust/privacy rating reflects the integrated treatment of security, privacy, explainability, bias, calibration, distribution shift, and lifecycle monitoring in Section 10. These ratings were author-assessed from the completed manuscripts using the stated criteria; they do not constitute independent quality scores.

### 1.4. Organization

Section 2 describes the review design, literature identification, evidence classification, and appraisal criteria. Section 3 establishes the connected diagnostic system boundary and data standards. Section 4 and Section 5 analyze how compute placement and acquisition quality affect diagnostic evidence. Section 6 and Section 7 examine learning and privacy-preserving collaboration. Section 8 then maps these components into explicit screening, diagnostic, prognostic, and decision-support pathways. Section 9 synthesizes domain evidence, and Section 10, Section 11 and Section 12 address reliability, datasets, validation, and translation. Section 13 and Section 14 restrict emerging directions and research priorities to those with a defensible diagnostic role before Section 15 and Section 16 present the review limitations and conclusions.

## 2. Review Design and Literature Selection

This study was designed as a structured integrative narrative survey. Its purpose was to connect heterogeneous engineering and clinical evidence across the complete AI-enabled IoMT diagnostic pathway rather than to estimate a pooled intervention effect. The literature search was conducted in Scopus from June 2026 through 14 August 2026. Five complementary search queries were used to cover (i) core AI–IoMT studies, (ii) diagnostic modalities and clinical functions within explicitly identified IoMT systems, (iii) distributed-computing and trust requirements, (iv) AI-supported diagnostic evidence from connected-health, wearable, biosensor, point-of-care, and remote-monitoring settings in which the term IoMT was not necessarily stated explicitly, and (v) clinical and external validation of connected diagnostic systems. The search logic was:

Q1: TITLE-ABS-KEY(("Internet of Medical Things" OR IoMT OR "Medical Internet of Things") AND ("artificial intelligence" OR "machine learning" OR "deep learning" OR "federated learning"))
Q2: TITLE-ABS-KEY(("Internet of Medical Things" OR IoMT OR "Medical Internet of Things") AND (diagnos* OR screen* OR detect* OR prognos* OR "clinical decision support" OR biosensor* OR wearable* OR "medical imaging" OR ECG OR EEG OR "point-of-care"))
Q3: TITLE-ABS-KEY(("Internet of Medical Things" OR IoMT OR "Medical Internet of Things") AND (edge OR fog OR cloud OR federat* OR privacy OR security OR explainab* OR blockchain OR "digital twin" OR "large language model"))
Q4: TITLE-ABS-KEY(("connected health" OR "connected healthcare" OR "remote patient monitoring" OR wearable* OR biosensor* OR "point-of-care" OR "mobile health" OR mHealth OR "edge healthcare") AND ("artificial intelligence" OR "machine learning" OR "deep learning" OR "federated learning") AND (diagnos* OR screen* OR "disease detection" OR "early detection" OR prognos* OR "clinical decision support" OR biomarker* OR "diagnostic accuracy" OR "clinical validation"))
Q5: TITLE-ABS-KEY(("Internet of Medical Things" OR IoMT OR "Medical Internet of Things" OR "connected health" OR "connected healthcare" OR "remote patient monitoring" OR wearable* OR biosensor* OR "point-of-care") AND (diagnos* OR screen* OR "disease detection" OR prognos* OR "clinical decision support") AND ("external validation" OR "prospective validation" OR "clinical validation" OR calibration OR "diagnostic accuracy" OR "clinical utility" OR "reference standard" OR "patient-level" OR "distribution shift" OR generalizab*))

The searches were limited to English-language journal articles, reviews, conference papers, and book chapters published from 2018 through the search cutoff. Earlier papers were retained only when needed to define an established technical or clinical standard. The five queries were intentionally overlapping because a publication could address more than one technological or clinical dimension. Their exports were merged, and duplicate records were removed deterministically using the Scopus Electronic Identifier (EID) as the primary unique key. Normalized DOI values were used as a secondary verification field; records lacking both identifiers were checked using title and publication year. Search results were supplemented by backward citation checking and targeted searches for reference standards and device-level benchmarks when a pathway-level claim could not be evaluated from the five queries alone.

### 2.1. Eligibility, Selection, and Bibliographic Verification

Publications were eligible when they contributed directly to at least one part of the sensing–computation–learning–diagnostic-action pathway: medical sensing or biosignal acquisition; edge, fog, or cloud placement; machine, deep, or federated learning; a defined screening, diagnostic, prognostic, or decision-support task; or security, privacy, explainability, interoperability, and validation requirements that affect diagnostic reliability. Purely generic IoT studies, papers without a medical or diagnostic connection, errata, preprints, retracted publications, and duplicate records were excluded. Studies were retained purposively to provide both pathway coverage and representative evidence, rather than to construct a fixed cohort for meta-analysis. Bibliographic records and DOI values were checked against the indexed export available at the cutoff date; online-first and conference records were retained only when their publication status and bibliographic metadata could be verified. Where a correction existed, the current publisher record was used and the corrected material was not counted as an independent study. Claims based only on information explicitly reported in an abstract were restricted to that level of detail.

Editorials were not eligible as empirical or review evidence. One guest editorial was retained solely as contextual background for framing security-assurance questions and was not used to support a quantitative result, diagnostic-performance claim, or clinical-effectiveness conclusion. The final thematic synthesis therefore contains 260 eligible evidence publications. In addition, the bibliography contains the one contextual editorial and four established clinical or engineering standards used to support interpretation of biosignal acquisition ranges, giving 265 cited items in total.

Because this is an integrative narrative survey rather than a systematic or scoping review, the iterative searches were not maintained as a single auditable screening cohort. The exact numbers of records identified before deduplication and screened at the title/abstract stage therefore cannot be reconstructed reliably and are not reported; this is acknowledged as a limitation of the review. No PRISMA flow denominator is claimed. Transparency is instead provided through the search blocks, cutoff date, eligibility rules, the final number of eligible evidence publications, evidence classes, and claim-specific citations.

### 2.2. Evidence Classification and Appraisal

Each empirical claim was interpreted according to the strongest evidence directly demonstrated by the cited study. The classes used throughout the synthesis are defined in Table 4. A result was not promoted to a higher class because it used a clinically named dataset or a deployable-sounding architecture.

For a purposively selected set of illustrative diagnostic studies, appraisal additionally considered four bias-sensitive domains: whether splitting occurred at patient level, whether the reference standard was clinically appropriate and independently established, whether external or temporal validation was performed, and whether calibration or a prespecified operating threshold was reported. The structured appraisal is presented later in the synthesis after the evidence-level framework has been established.

“Not reported” was treated as uncertainty, not as evidence that the safeguard was absent.

Table 2 uses a separate scope-coverage rubric rather than an evidence-quality score. “Deep” requires a dedicated section, treatment of multiple subtopics, and explicit comparison or limitations within the rated dimension. “Partial” indicates substantive discussion that is secondary to the survey’s organizing focus or covers only part of the dimension. “Limited” indicates an isolated mention, a brief subsection, or no comparative synthesis. Ratings were applied to the published survey as a whole and do not rank methodological quality. The author team reviewed the completed matrix collectively and reconciled differences by discussion; no inter-rater coefficient was calculated because the categories describe scope rather than risk of bias. The surveys were selected because their declared focus intersects one or more of the six comparison dimensions and together they represent application-, technology-, security-, federation-, and system-oriented review perspectives.

## 3. Background: From Connected Devices to Intelligent Healthcare

The IoMT inherits sensing, communication, processing, and application layers from the wider IoT, but medical use changes the consequence of failure at every layer. A disconnected consumer sensor loses convenience; a disconnected medical sensor can suppress an alarm or interrupt a therapeutic loop. A useful system boundary must therefore include the device, network, analytic service, clinical recipient, and action that follows the output. This distinction is more informative than defining IoMT as a collection of connected medical devices because it exposes where safety, privacy, and semantic meaning can be lost [2].

Healthcare 4.0 accounts connect this end-to-end system to precision medicine, remote diagnosis, and continuous observation. Their main contribution is architectural: value arises when sensing, analytics, and care delivery are coordinated, not from adding connectivity to an otherwise unchanged workflow. However, many benefits described at this level remain capabilities rather than demonstrated clinical outcomes, so later sections separate technical feasibility from evidence of utility [22]. Extending this trajectory, Healthcare 5.0 moves the architecture toward human-centered services supported by AI and next-generation communication. The useful part of that vision is its insistence that automation remain subordinate to patient and clinician needs. Claims involving 6G or pervasive autonomy should nevertheless be treated as design targets until latency, reliability, and safety are verified in the intended clinical environment [23].

### 3.1. A Layered Reference Model

The four-layer model—perception, network, processing, and application—is useful only if the interfaces between layers are explicit. Sampling policy changes traffic and battery use; compression changes signal morphology; compute placement changes latency and exposure; and the application layer determines whether an output is merely displayed or becomes a clinical intervention. A security-and-management plane must therefore cross the whole stack rather than protect only cloud storage. Technical surveys consistently identify this coupling as the defining implementation difficulty, although they provide limited evidence that one universal architecture can serve every medical pathway [24].

An applications-first view reaches the same conclusion from the opposite direction. Wearables, implantables, remote monitoring, and hospital systems impose different limits on energy, response time, biocompatibility, and acceptable autonomy. Treating them as interchangeable IoMT endpoints hides the engineering decisions that determine whether a system is safe and maintainable. The architecture should consequently be derived from the intended clinical action, not selected before that action is specified [25].

### 3.2. Three Waves of Capability

Three overlapping capability waves clarify how the design problem has changed. Telemetry transported measurements; connected analytics added remote storage and dashboards; embedded intelligence now performs quality control, inference, and sometimes adaptation close to the patient. Hospital-oriented IoT work shows that connectivity can improve asset visibility and service coordination, but it does not by itself establish diagnostic validity or better patient outcomes [26].

Wearable-device surveys document the second transition: measurement moved from episodic encounters to continuous streams. This increases temporal coverage while also introducing adherence, placement, drift, and missing-data problems that are uncommon in supervised bedside acquisition. The relevant advance is therefore not simply more data, but the ability to recognize when continuously collected data are trustworthy enough for interpretation [27]. Within that continuous setting, deep-learning integration marks the third wave because feature extraction and classification can be learned jointly from large sensor streams. The gain is strongest for high-dimensional signals, but the resulting models are harder to audit, compress, and transfer between devices. A survey that reports only diagnostic accuracy consequently misses the deployment cost created by model size and opacity [28].

Real-time monitoring studies reveal a second limitation: a model can be accurate offline yet clinically late when acquisition windows, transmission, queueing, and human response are included. The evidence should therefore report end-to-end delay and failure behavior, not inference time alone [29]. This timing constraint matters because the proposed move toward value-based care is credible only when a learned output changes a decision, workload, or outcome. Most AIoMT studies remain at retrospective prediction or prototype monitoring, so “value” should be treated as a hypothesis that requires prospective workflow evaluation rather than inferred from classification performance [30].

A learn–predict–monitor–detect–correct loop provides a more operational formulation because it includes observation after the model acts. Its weakness is that correction is safe only when authority, uncertainty, and rollback are specified. In critical care, a closed loop requires substantially stronger evidence than a system that merely notifies a clinician [31]. At the broader systems level, AIoT syntheses are useful for showing that sensing and learning are becoming inseparable, but broad convergence arguments do not decide where computation belongs or how much autonomy is acceptable. Those questions must be resolved at the level of the clinical pathway and device budget [32]. At the implementation level, experimental reviews of smart-healthcare development add implementation examples, yet heterogeneous tasks and validation protocols prevent their reported metrics from being compared directly. The defensible synthesis is architectural—which functions are distributed across the stack—rather than a ranking of incompatible accuracy values [33].

Broader healthcare-technology surveys place intelligent sensing alongside imaging, robotics, telemedicine, and decision support. This breadth is useful for locating IoMT within digital health, but it also reinforces the need to keep the scope on systems in which networked medical measurements materially participate in the decision [34].

### 3.3. Standards, Interoperability, and the Data Substrate

Interoperability is an input-quality requirement for AI, not an administrative feature. Transporting a number without its unit, body site, sampling context, device identity, or patient association produces syntactic connectivity but not clinically usable data. Fragmented formats and semantics therefore limit both model training and safe reuse across institutions [2].

Next-generation-network surveys describe very low latency and massive connection density as enablers of remote and distributed healthcare. These capabilities are relevant to dense monitoring and time-sensitive offload, but radio targets should not be reported as clinical latency: sensing windows, preprocessing, queues, application logic, and human response may dominate the path [35]. The same network expansion also exposes a privacy trade-off. Greater sensing density and localization capability can improve context awareness while increasing the resolution at which individuals and behaviors can be inferred. Security and minimization must therefore be designed with the communication service rather than added after deployment [36]. Beyond transport, a medical digital twin requires more than a dashboard synchronized with sensor readings. It must preserve identity, time, provenance, uncertainty, and a defensible relationship between observed patient state and simulated response. Industry-4.0 reviews explain the integration of AI, IoT, and patient models, but the clinical claim remains bounded by the evidence used to validate the twin’s predictions [37].

Sensor-enabled twin research broadens this idea to smart environments, where contextual measurements can complement body-worn data. The benefit is richer state estimation; the risk is incorrect attribution when multiple people, devices, or spaces contribute observations. Reliable identity resolution is therefore part of the model, not merely a networking detail [38]. These requirements establish what information must be preserved; the next design decision is where that information should be processed.

## 4. The IoMT Architecture: A Sensing–Edge–Fog–Cloud Continuum

**Figure 2:**
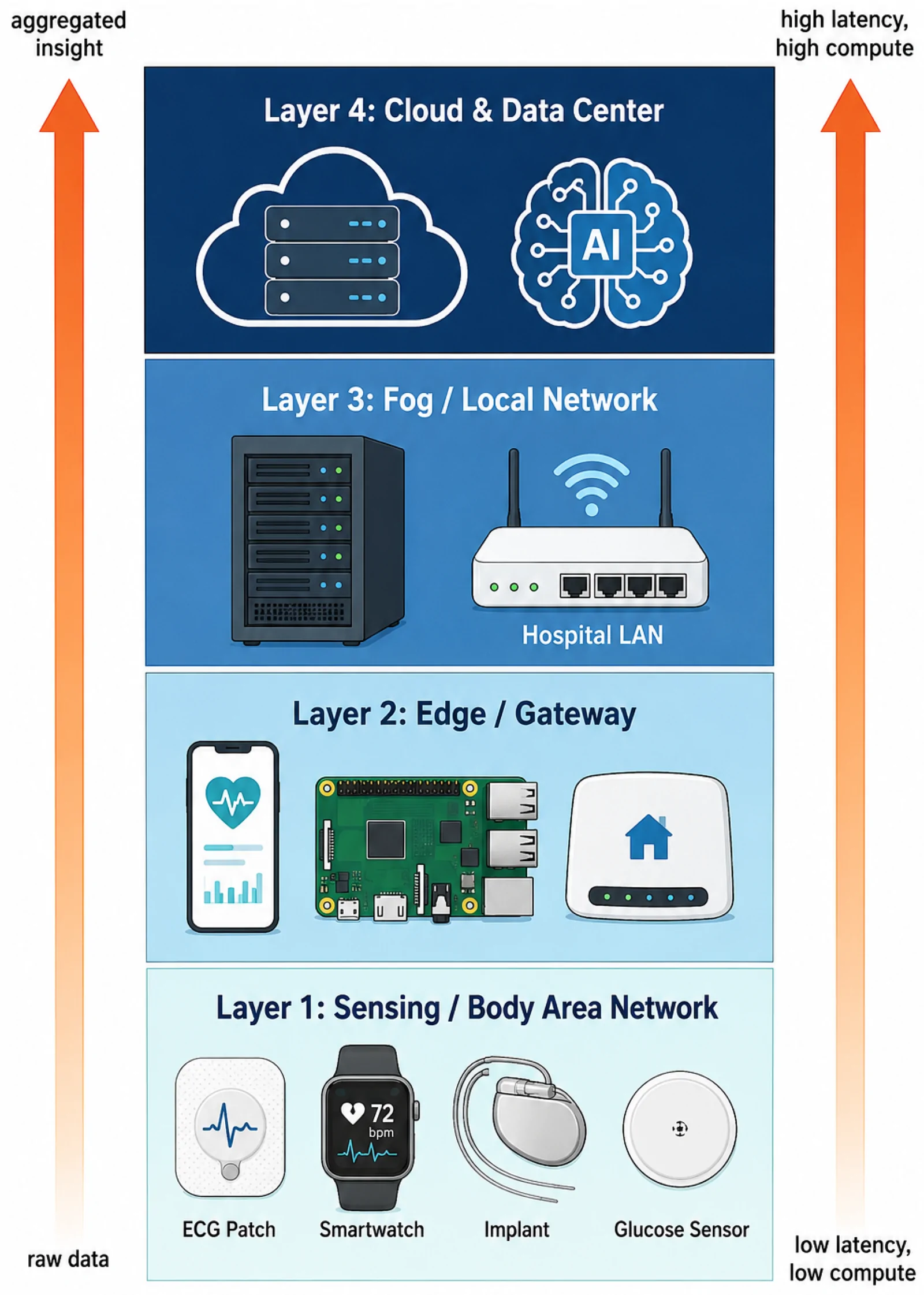
The sensing–edge–fog–cloud continuum. Moving upward increases available compute but also latency; a core design task is placing each analytic function at the tier that satisfies its timing, energy, and privacy constraints.

A useful way to reason about any intelligent IoMT system is to picture a vertical continuum, illustrated in Figure 2, along which both data and computation move: sensing and body-area devices at the bottom, edge gateways above them, fog nodes at the local network, and the cloud at the top. Each tier trades latency for capability—the sensing device answers in milliseconds but has almost no memory, whereas the cloud has effectively unlimited resources but answers only after data traverses the network. The central architectural question is therefore not *whether* to use the cloud but *which computation belongs at which tier*. Pazmiño-Ortiz et al. frame exactly this as the cross-cutting problem of workload placement across the device–edge–fog–cloud continuum, and note that most prior reviews examine one layer in isolation rather than the placement decision itself [39].

### 4.1. The Four Tiers and What Belongs on Each

Latency-critical inference usually belongs near the patient, whereas population training and durable longitudinal storage favor the cloud. Comparative evidence from chronic-disease frameworks supports fog placement for preprocessing and streaming inference, but it also shows why isolated accuracy values are misleading: some controlled cardiovascular studies report values above 99%, while small datasets, imbalance, curated benchmarks, and leakage can produce optimistic estimates. Multi-fog provisioning further reduces delay at the cost of energy and operational complexity [40].

An IoT–fog diagnostic framework for diabetes and cardiovascular disease demonstrates the appeal of near-source classification, reporting maximum accuracies of 97.26% and 96.16%, respectively. Because the abstract reports validation on medical records without describing external-site or prospective testing, these results establish comparative technical performance rather than transportability to underserved populations or clinical benefit [41]. That placement benefit can also be evaluated as a queueing problem instead of asserted qualitatively. A random-forest segmentation method coupled with a stochastic service model reported 92% classification accuracy and a 56% latency reduction in simulation. The joint reporting of prediction and system response is valuable, but simulation-based latency does not capture device failures, network variability, or clinician response in deployment [42].

Cloud–fog monitoring remains useful when local nodes handle urgent classification and the cloud provides coordination and storage. A heart-disease system reported approximately 97% across several classification metrics, but its simulation-based evaluation cannot establish real-time clinical performance despite the system’s label. The result supports architectural feasibility, not outcome effectiveness [43].

Table 5 summarizes the characteristic role and typical intelligence placed at each tier. The latency entries are illustrative observations from representative implementations or models, not universal tier guarantees; workload, radio access, queueing, routing, congestion, hardware, and the sensing-window duration can dominate end-to-end delay.

### 4.2. Edge Intelligence: Bringing the Model to the Data

The architectural case for edge intelligence rests on three recurring failures of cloud-only designs: urgent decisions inherit network delay, centralization enlarges the privacy boundary, and loss of connectivity can disable service. A critical review finds that the field has responded with compact models and edge gateways, but evaluation remains dominated by small or single-site datasets and fragmented security mechanisms. Moving inference is therefore not equivalent to making it dependable [47].

A systematic review of 37 edge-healthcare studies finds broad use of support-vector machines, clustering, deep learning, and federated learning. The consistent benefits are reduced latency and reduced raw-data movement; the consistent limitations are device resources, security, and absent standard protocols. This evidence supports edge placement as a design option, not as a universally superior tier [48]. The same edge logic appears beyond bedside monitoring: pandemic-resilient smart-city work illustrates a boundary case in which edge and cloud components jointly process images for distancing, mask use, and contact tracing. The design shows how local processing can support population-scale response, but its tasks and public-space assumptions differ substantially from physiological monitoring, so its performance should not be generalized to bedside IoMT [49].

TinyML makes persistent local inference possible by constraining model memory and computation to microcontrollers and wearables. Its strongest medical value is continuous screening when communication is intermittent or expensive. Its central limitation is not only reduced capacity: updates, calibration, security, and evidence must remain manageable over the device lifetime [50]. Within clinical sensing, EEG data reduction shows why preprocessing belongs in the placement decision. Compression and feature extraction can reduce transmission volume and expose class-relevant structure before data reach the fog. The design is safe only when reconstruction or task-level degradation is quantified, because a smaller representation may discard morphology required by a later clinical question [51]. A stronger end-to-end example comes from blood-glucose prediction. An attention-based evidential recurrent model was embedded in a low-power wearable and evaluated on three clinical datasets comprising 47 people with type-1 diabetes; hardware-in-the-loop trials with ten virtual adults then tested predictive low-glucose management. The system reduced hypoglycemia in the simulated control experiment, but the small clinical cohort and in-silico intervention stage still leave prospective outcome validation open [52].

Communication-efficient distributed AI addresses the cost created when models rather than raw data move among edge nodes. Update compression and selective exchange can reduce bandwidth, but they also change convergence and may amplify heterogeneity across devices. Reported communication savings should therefore be paired with accuracy, fairness, and time-to-convergence under realistic links [53]. The same near-source logic extends from signals to text: fog processing can protect unstructured medical text before cloud use. A CNN–BiLSTM sanitization model reported 92% F1 and improved utility preservation for medical-entity recognition, demonstrating that privacy transformation can be integrated with near-source analytics. Because successful entity removal is not identical to resistance against re-identification, the privacy claim still requires an explicit adversary and attack evaluation [54].

Security-focused edge syntheses correctly treat privacy, model robustness, and resilience as coupled properties.

The guest editorial retained in this survey is used only to frame assurance questions that empirical systems should answer; it is not treated as comparative experimental evidence or as support for a quantitative or clinical-effectiveness claim [55].

Beyond privacy, cloud–IoT resilience discussions add failure prediction, load balancing, and self-recovery to the usual latency argument. Their healthcare examples remain conceptual, but they identify an important systems requirement: local inference is valuable only if the surrounding service can detect faults and degrade safely [56].

A cardiac-monitoring implementation demonstrates that interpretability and security can fit within an edge budget. It reports 8.1-ms inference on an ESP32 with a 350-KB footprint, AUC 0.985 on MIT–BIH, and lower but still strong zero-shot AUCs on PTB-XL and Chapman. The external-dataset decrease is scientifically useful because it reveals distribution shift; the remaining limitation is that benchmark generalization and generated explanations do not substitute for clinician or prospective evaluation [44].

#### Formalizing the Placement Decision

The latency-versus-accuracy trade-off can be stated precisely. Consider a deep model whose layers are indexed 1,…,L and partitioned at a split point *s*, so that layers 1,…,s run on the device and s+1,…,L run in the cloud. Let $t_{i}^{\mathrm{dev}}$ and $t_{i}^{\mathrm{cl}}$ denote the execution time of layer *i* on the device and in the cloud, let $d_{s}$ be the size (in bits) of the intermediate representation that must be transmitted at the cut, and let *R* be the uplink rate. The end-to-end inference latency is

(1) $$T\left(s\right)=\underset{\text{on-device}}{\underset{︸}{\sum_{i=1}^{s}t_{i}^{\mathrm{dev}}}}+\underset{\text{serialization}}{\underset{︸}{\frac{d_{s}}{R}}}+\underset{\text{queueing},\;\text{jitter},\;\text{multi-hop}}{\underset{︸}{q_{s}+j_{s}+h_{s}}}+\underset{\text{in-cloud}}{\underset{︸}{\sum_{i=s+1}^{L}t_{i}^{\mathrm{cl}}}},$$

Here, $q_{s}$ is the queueing delay at the device, gateway, fog node, or service; $j_{s}$ represents stochastic network variation; and $h_{s}$ collects propagation, forwarding, and processing delay across one or more fog or network hops. These quantities are random and path dependent, so placement should be optimized against a latency distribution or tail requirement rather than a mean-only point estimate [42]. The optimal split point is $s^{★}=\arg\min_{0\leq s\leq L}T\left(s\right)$ subject to the device energy budget $E\left(s\right)\leq E_{\max}$.

Equation (1) makes the two degenerate cases explicit: s=L is pure on-device inference (no transmission term, but the device pays for every layer), while s=0 is the cloud-only baseline (the whole input is shipped and $d_{0}$ is largest). It also shows why quantization is so effective at the edge: compressing activations from $b_{0}$ to $b_{q}$ bits scales the transmitted volume as

(2) $$d_{s}^{\;q}=d_{s}\;\frac{b_{q}}{b_{0}},$$

which shrinks the middle term of (1) and can move $s^{★}$ deeper into the device, precisely the behavior the communication-efficient and data-reduction methods discussed above are designed to exploit. The accuracy cost of (2)—the drop incurred when $b_{q}$ is small—is the price paid on the “accuracy” side of the trade-off, and bounding it is the objective of the compression techniques discussed in Section 6.

### 4.3. Enabling Networks and Emerging Architectural Motifs

Beneath the compute continuum lies a communication substrate whose rate, outage behavior, and energy cost bound the functions that can be offloaded. On-device neural-network deployment responds with quantization, pruning, knowledge distillation, and architecture search. These techniques reduce memory and latency, but every reduction must be evaluated on the target processor and medical task because compression ratios alone do not reveal accuracy loss or worst-case timing [57].

Self-powered triboelectric transducers combine energy harvesting with tactile sensing and emerging neuromorphic interfaces. Their potential is strongest for long-lived wearable or implantable acquisition, while stability, device compatibility, scaling, and cost remain unresolved. At present they support a technology trajectory rather than evidence for routine clinical deployment [58]. Patient-centered digital-twin frameworks integrate IoT measurements with AI-based state estimation to support personalized and predictive care. Their main unresolved issues include heterogeneous longitudinal data, clinical validation, responsibility for incorrect recommendations, privacy, and security. These are not peripheral governance concerns: they determine whether a continuously updated model can be trusted as a representation of an individual [59].

AI-generated patient and organ twins extend static models toward predictions of future health and in-silico intervention comparisons. Their value depends on longitudinal linkage across wearables, clinical records, and omics; their risk is that an apparently individualized prediction may inherit population bias or unobserved confounding. Seamless infrastructure integration and validation remain prerequisites [60]. Yet maturity remains uneven: migraine illustrates the gap between a plausible twin and an evaluated one. Continuous journals and wearable streams could support personalized trigger and treatment modeling, but the available work is primarily a proposed construction derived from adjacent neurological literature. It identifies a credible research direction without demonstrating improved migraine outcomes [61]. By contrast, UbeHealth provides an earlier proof of concept for coordinated cloud and edge operation. It uses traffic prediction, caching, routing, and malicious-traffic detection across four layers and evaluates the framework with three public datasets. This validates component behavior but does not establish clinical utility, reinforcing the distinction between network optimization and healthcare effectiveness [62]. Table 6 contrasts the communication technologies that carry IoMT data and the roles they play.

## 5. The Sensing Layer: Wearables and Biosignals

**Figure 3:**
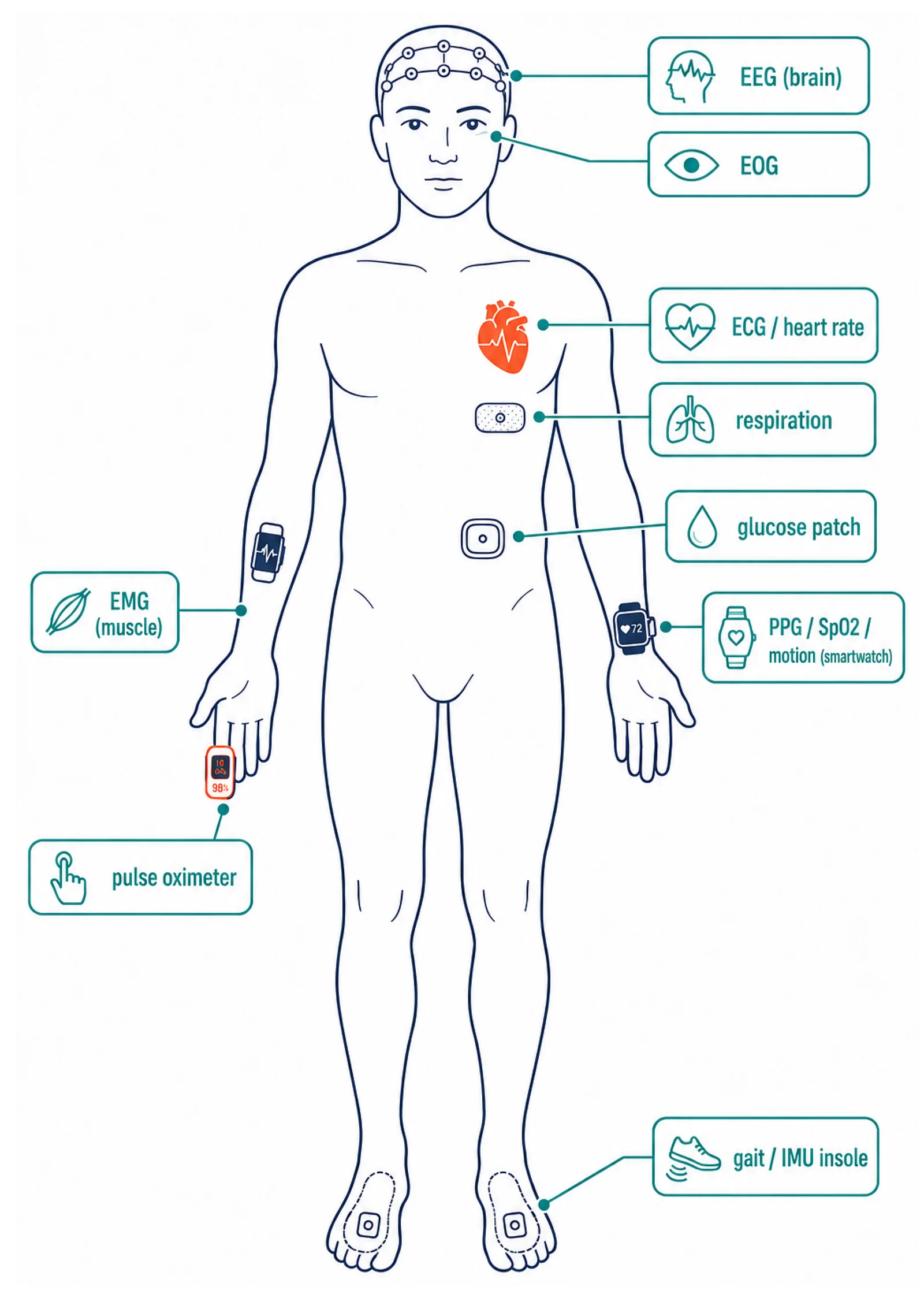
The sensing layer organized by biosignal and body placement. Signal modality, sampling rate, and noise characteristics determine which analytic methods are feasible downstream.

The compute continuum determines where information is processed, but the sensing interface determines whether useful information exists in the first place. Every intelligent decision in the IoMT begins with a physical measurement, and no algorithm can recover information a poor sensor never acquired. The sensing layer spans body-worn and implantable devices and the biosignals they capture, organized in Figure 3 by signal type and body placement and summarized in Table 7.

### 5.1. A Taxonomy of Biosignals

The dominant IoMT biosignals are electrophysiological (ECG, EEG, EMG, and EOG), optical (PPG), inertial, and electrochemical. Each creates a different learning problem because its rate, morphology, artifact mechanisms, and clinically relevant timescale differ. Single-lead ECG is attractive for continuous atrial-fibrillation screening, but reduced lead count sacrifices spatial information and makes signal-quality control central to any claim of dependable detection [63].

The broader arrhythmia literature confirms that portability and automated interpretation have advanced faster than real-world validation. Common gaps include dependence on curated databases, inconsistent preprocessing, and limited evidence under motion or device change. Continuous monitoring should therefore be evaluated by patient-level sensitivity and false alarms per unit time, not only beat-level accuracy [64]. Self-supervised learning addresses the scarcity of labeled ECGs without pretending that unlabeled data solve every problem. Direct lead assignment reported a four- to eightfold reduction in label dependence and real-time execution of at most 388 ms per 10-s record on a Raspberry Pi Zero 2 W. This is evidence of compute feasibility and label efficiency; it does not establish robustness across hospitals unless the pretraining and test populations are independently separated [46].

A one-channel seizure predictor shows how portability and temporal modeling can be combined. A CNN–LSTM selected after model comparison achieved 0.9691 accuracy, 0.9753 sensitivity, and 0.9630 specificity on CHB–MIT, while a Raspberry-Pi edge device produced an alert with 163-ms processing latency. Because the evaluation relies on one public database, the result supports a wearable prototype but not yet cross-device or prospective seizure forecasting [45]. Multi-channel EEG emotion recognition benefits from modeling both long-range dependencies and spatial relations among electrodes. A dynamic graph-convolution transformer reported accuracies near 99% on SEED and DEAP. Such values on established affective datasets are technically strong, but predefined laboratory labels and subject-dependent patterns limit their interpretation as evidence for clinical mental-health diagnosis [65].

EEG distribution shift is visible across people, acquisition times, and devices. Hierarchical domain adaptation explicitly learns shared representations across those domains and outperformed comparison methods in two epilepsy-classification experiments. The approach addresses a real deployment failure mode, although the abstract does not report prospective testing or uncertainty when adaptation encounters an unseen domain [66].

### 5.2. Sensors, Materials, and the Energy Constraint

Digitally connected point-of-care biosensors shorten the path from measurement to interpretation, but connectivity does not correct analytical drift, cross-reactivity, or poor calibration. The strongest designs therefore report both assay performance and the behavior of the digital pipeline that carries the result [67].

Wearable and implantable BioMEMS broaden continuous monitoring and therapy while introducing packaging, biocompatibility, fouling, and replacement constraints. These lifecycle properties can dominate nominal sensor sensitivity, particularly for implants that cannot be serviced easily [68]. Biosensor taxonomies are valuable when they connect transduction principles to interference and intended use. Classifying a device as electrochemical, optical, piezoelectric, or field-effect based is only the first step; clinical interpretation also requires a reference method, operating range, and stability under the target sample matrix [69].

Deep learning can assist nanozyme-based sensing by relating material design and complex response patterns to analyte prediction. This shifts part of selectivity from chemistry to computation, which may improve sensitivity but also makes dataset composition and recalibration part of the assay’s validation burden [70]. Self-powered sensor systems address the adherence and maintenance cost of frequent charging. Combining harvested energy with local AI is attractive for persistent monitoring, but intermittent supply can change sampling and inference schedules. Energy autonomy should therefore be reported as a duty cycle under realistic excitation, not as peak harvested power [71]. Mechanoluminescent materials convert mechanical input into optical output and may support passive or low-power sensing. Current evidence is strongest at the materials and device level; stability, repeatability, and clinically meaningful integration remain more important than adding an AI label to the readout [72].

Wearable-AI reviews show that local inference can reduce latency and data movement, but they also identify memory, battery, personalization, and security as coupled constraints. Model accuracy measured off-device is consequently insufficient evidence that the same model is viable on a wearable [73]. A wireless vital-sign implementation illustrates what an integrated evaluation can report. Using SpO_2_, heart rate, and temperature, it compared KNN, SVM, and SGD classifiers and reported accuracies of 92%, 94%, and 97%, with the strongest AUROC for SGD. Validation against a health-scoring system supports technical classification, but the abstract does not establish prospective deterioration detection or outcome benefit [74].

Industry-5.0 narratives place intelligent wearables within personalized, human-centered care. Their useful contribution is to broaden evaluation beyond the sensor to usability and service integration; their forward-looking claims should not be confused with evidence that a specific device improves care [75]. Cardiovascular wearable reviews consistently locate the deployment barrier in unsupervised field use, where motion, placement, skin contact, adherence, and population shift interact. A model that performs well on clean recordings may therefore fail precisely during the activities continuous monitoring is intended to capture [76].

AI-enhanced electrochemical sensing can model nonlinear relationships among response curves, interferents, and biomarker concentrations. Its advantage is most credible when training and testing are separated by patient or batch and when predictions are compared with an accepted reference assay [77]. Electrochemical and optical pathogen sensors expanded rapidly during COVID-19, offering faster and more portable assessment. Their IoMT value depends on preserving sample identity, quality-control status, and uncertainty from the assay to the remote recipient; wireless reporting alone does not establish diagnostic validity [78]. Two-dimensional materials support highly integrated intelligent biosensors and motivate the hospital-on-chip concept. The concept joins sensing, processing, and communication at small scale, but its clinical readiness is bounded by fabrication reproducibility, biofouling, calibration, and validation with real specimens [79].

HbA1c point-of-care testing demonstrates the maturity gradient from laboratory method to connected assay. A review spanning electrochemical, optical, mass-based, colorimetric, fluorescent, and chip techniques emphasizes standardization and technology readiness. AI and mobile integration may automate interpretation, but they cannot compensate for an analytically unvalidated measurement [80]. A federated edge architecture co-designs sensing and protection across ECG patches, glucose monitors, neurostimulators, and cardiac devices. It reported 96.2% classification accuracy, 36% lower end-to-end latency, and 29% lower communication cost than two simulated baselines. Because the evaluation was conducted using synthetic multimodal data in a simulated environment, these results establish comparative architectural feasibility rather than clinical or hardware performance [81].

### 5.3. From Raw Signal to Model-Ready Data

Between acquisition and inference, filtering, artifact control, segmentation, and representation determine which clinical information survives. A multi-tier arrhythmia design adapts AI modules to heterogeneous node hardware and reported 99.3% maximum accuracy in simulation. The architecture addresses compatibility and selective escalation, but simulated emergencies do not establish timing or reliability on physical devices and patients [82].

TinyML provides stronger device-oriented evidence when preprocessing, inference, memory, and power are measured together. An optimized ECG anomaly detector reported 92.3% accuracy and 0.024 mW power on Raspberry-Pi and Arduino-class platforms. The result shows a favorable resource trade-off, although continuous field testing is still needed to quantify motion artifacts and false alarms [83]. An ensemble combining DenseNet201 features with SAE, GRU, and LSTM classifiers reported 98.7% accuracy, 97.5% sensitivity, and 99.1% specificity on a benchmark ECG dataset. Optimization improves performance under the chosen benchmark, but the complexity of the pipeline and absence of device-level measurements weaken its claim to IoT readiness [84]. Ear-EEG reduces obtrusiveness while a superlet representation and modified vision transformer model time–frequency structure. An average 92.39% accuracy on untrained datasets is encouraging, but emotion labels and wearable fit remain sources of uncertainty that must be tested in naturalistic use [85].

Neonatal sleep staging illustrates the value of minimizing channel count. A single-channel pipeline extracted 490 multidomain features and used stacked classifiers, reaching 90.37–94.88% accuracy across binary tasks with k-fold and leave-one-subject validation. The subject-level analysis strengthens the evidence, while the small second cohort of 19 neonates limits precision for broader deployment [86]. Encrypted EEG classification converts signals into spectrograms, applies chaotic transforms, and feeds transfer-learning CNNs. Evaluation on CHB–MIT showed favorable performance relative to several CNNs, but encryption against disclosure is different from authenticated integrity and resistance to manipulation; the security claim should remain limited to the mechanisms actually tested [87].

A quantum-blockchain seizure framework reported up to 96.78% classification accuracy and analytical resistance to several quantum attacks. The work is forward-looking, but claims of exponential speedup and attack resistance require physical implementation and explicit resource accounting before they can guide near-term IoMT deployment [88]. Multimodal ECG–PPG biometrics can authenticate access continuously while homomorphic encryption protects cloud processing. A study of 20 users reported 100% identification under several seated configurations and feasibility on low-end processors. The limited cohort and controlled posture make this proof of concept insufficient for population-scale identity assurance [89].

An EEG framework for major depressive disorder combines S-transform features, graph convolution, and a transformer, reporting 99.92% and 99.68% accuracy on HUSM and MODMA. Testing two datasets is stronger than a single-benchmark result, but retrospective EEG classification does not yet demonstrate real-time monitoring, diagnostic utility, or robustness to clinical comorbidity [90]. An end-to-end ECG monitoring system compared random forest, CNN, and KNN models connected to a Polar H10 sensor. KNN produced class-dependent accuracies from 81% to 97%, revealing that aggregate accuracy would hide weaker arrhythmia classes. This class-specific reporting is useful, although validation on the deployed acquisition pathway remains necessary [91]. The consistent lesson is that signal conditioning, split discipline, and device-level evaluation are not preliminary details. They define what the downstream model has actually learned and whether its output remains valid after the sensor leaves the laboratory.

**Table 7:** Principal biosignals in the IoMT and the processing considerations they impose. Rates are representative acquisition ranges rather than universal prescriptions; the intended diagnostic bandwidth, device specification, and applicable clinical or engineering standard govern the final setting.

Signal	Source/Measurement	Typical Rate	Dominant Noise	Common AI Task
ECG	Cardiac electrical activity	100–500 Hz [92]	Baseline wander and motion [63,64]	Arrhythmia/AF detection
PPG	Optical blood-volume change	25–125 Hz [93]	Motion artifact [93]	Heart rate, SpO_2_, BP estimation
EEG	Cortical electrical activity	128–512 Hz [94]	Muscle and eye-blink artifact [94]	Seizure, emotion, fatigue
EMG	Muscle electrical activity	Application-specific; commonly kHz-scale [95]	Crosstalk and motion [95]	Gesture, rehabilitation
IMU/accel.	Motion and orientation	Application-specific; commonly tens to hundreds of Hz [16]	Placement variation [16]	Activity, gait, fall detection
Electro-chemical	Analyte concentration	Assay- and response-time dependent [80]	Drift and temperature [80]	Glucose, biomarker sensing

## 6. Machine Learning and Deep Learning in the IoMT

**Figure 4:**
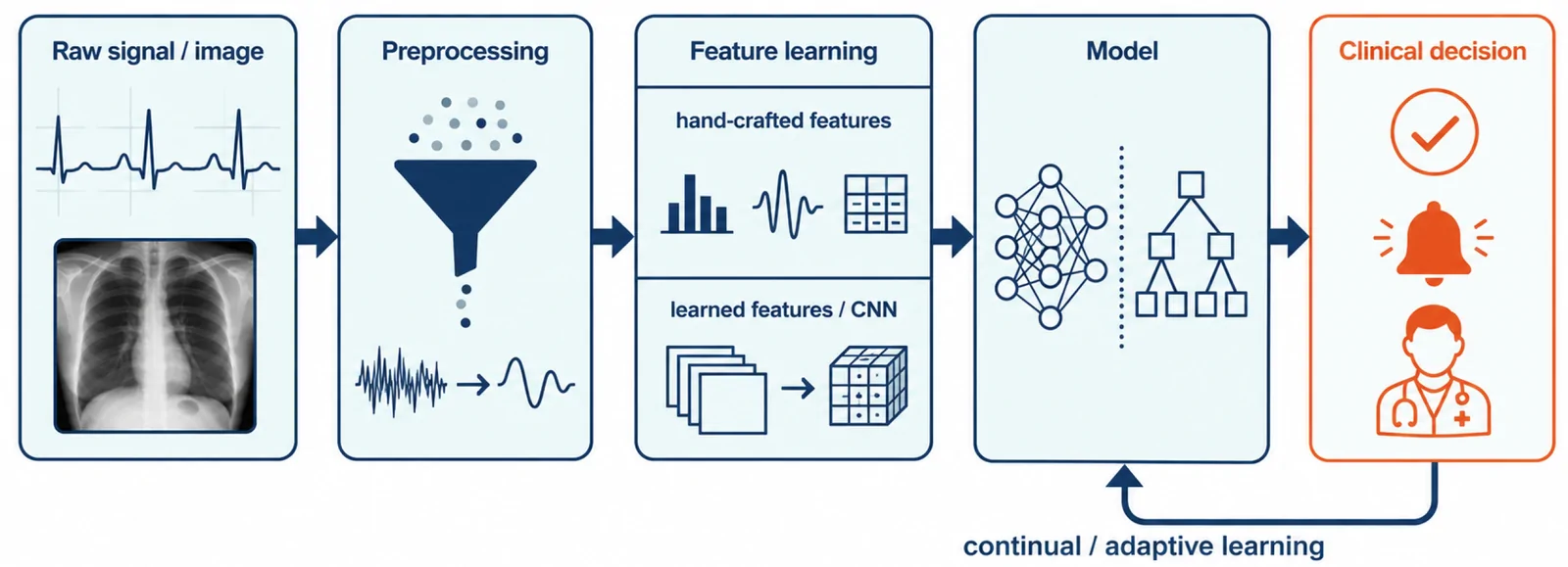
The shared analytics pipeline in the IoMT. Classical machine learning and deep learning differ chiefly in the feature-learning stage; both feed a decision that can, in adaptive systems, refine the model over time.

With clean signals in hand, the central act of the intelligent IoMT is turning them into decisions. Two families of methods do this work—classical machine learning on engineered features, and deep learning on raw data—and knowing when to use each is one of the most practically important skills in the field. Figure 4 shows the pipeline both families share, and this section examines each in turn, the training pathologies peculiar to medicine, and the compression techniques that make deep models deployable.

### 6.1. The Supervised Learning Problem, Stated Formally

Both families of methods solve the same underlying problem, and stating it once makes the later trade-offs precise. Given a dataset $D={\left\{\left(x_{j},y_{j}\right)\right\}}_{j=1}^{n}$ of physiological inputs $x_{j}$ and clinical labels $y_{j}$, a model $f_{\theta }$ with parameters θ is trained by empirical risk minimization,

(3) $$\theta^{★}=\arg\min_{\theta }\;\frac{1}{n}\sum_{j=1}^{n}\ell \;\left(f_{\theta }\left(x_{j}\right),y_{j}\right)+\lambda \;\Omega \left(\theta \right),$$

where *ℓ* is a loss, Ω a regularizer, and λ its weight. For the classification tasks that dominate the IoMT—arrhythmia present or absent, seizure imminent or not—*ℓ* is the cross-entropy

(4) $$\ell_{\mathrm{CE}}\left(p,y\right)=-\sum_{c=1}^{C}y_{c}\log p_{c},\;p_{c}=\frac{e^{z_{c}}}{\sum_{k=1}^{C}e^{z_{k}}},$$

with *z* the model’s logits over *C* classes. Two properties of (3) and (4) explain much of what follows. First, because pathological events are rare, the class prior is highly skewed, so the average loss in (3) is dominated by the majority (healthy) class unless it is reweighted—the formal reason accuracy misleads and why the imbalance-aware training of Section 6 and the metrics of Section 11 are necessary. Second, (3) assumes training and deployment data are drawn from the same distribution; when a compressed model trained at one hospital meets another’s sensors and case mix, that assumption fails, which is the “domain shift” half of the model-size-versus-robustness trade-off examined below.

### 6.2. Classical Machine Learning and Feature Engineering

Classical supervised learning—logistic regression, tree ensembles, and support-vector machines—remains central because it can train on modest tabular datasets and execute within constrained gateways. Case-based syntheses show that these methods often remain competitive when variables already have clinical meaning, but they also inherit bias, missingness, and documentation practices from the underlying records. Interpretability is useful only when the features themselves are valid and the decision threshold is clinically justified [96].

Distributed medical-data pipelines favor classical models when training data are fragmented and inference must be auditable. Their lower computational burden simplifies deployment, yet cross-site preprocessing and semantic alignment may matter more than the choice among standard classifiers. A model cannot compensate for incompatible definitions of the same clinical variable [97]. Healthcare-4.0 surveys describe supervised learning as a way to reduce manual diagnostic effort. The defensible role is usually screening or decision support rather than autonomous diagnosis because retrospective labels encode clinician behavior and may not represent a reproducible reference standard [98].

The broader interaction between AI and distributed systems supports proactive monitoring, but it also exposes a systems dependency: a transparent model can still be unsafe if the sensor, data path, or alert workflow is unreliable. Classical methods are therefore preferred when their simpler behavior helps satisfy the complete service requirement, not merely because they are smaller [99].

### 6.3. Deep Learning Architectures for Medical Data

Deep learning changes the pipeline by learning representations jointly with the prediction task. This is valuable for images and waveforms, but it also ties performance to dataset scale, label quality, and the match between training and deployment devices. IoT integration increases access to data without automatically increasing its clinical validity [28].

Biomedical big-data syntheses show why deep models thrive on heterogeneous high-dimensional inputs. They also reveal a reproducibility problem: architecture novelty is often emphasized more than patient splits, calibration, and external validation. Those evidence properties determine whether a reported gain is portable [100]. A hybrid skin-lesion system combines segmentation and classification within an IoMT setting. The clinically relevant question is not only lesion accuracy but whether images come from independent patients and devices and whether uncertain cases are referred. Without those details in the abstract, the work supports algorithmic feasibility rather than autonomous screening [101].

Cloud-delivered CNN lung-cancer diagnosis illustrates how computation can be made ubiquitously accessible. The cloud solves capacity and availability of inference, not dataset bias or diagnostic confirmation; service reach should therefore be reported separately from clinical performance [102]. Optimized CNN lung-cancer work links early diagnosis to IoMT acquisition. Optimization may improve benchmark classification, but unless the same acquisition path is used in evaluation, the IoMT component remains an architectural wrapper around an imaging model [103].

A hierarchical CNN for pancreatic-tumor diagnosis similarly demonstrates image-analysis integration with a connected platform. Tumor detection in curated images does not establish earlier detection in an eligible clinical population, where prevalence, spectrum, and reference labels differ [104]. Brain-tumor surveys confirm the dominance of convolutional architectures and the burden of manual interpretation. Their central unresolved issue is evidence comparability: studies use different modalities, tumor classes, preprocessing, and split units, so headline accuracies should not be ranked without task alignment [105]. Ophthalmic AI spans screening, segmentation, prognosis, and personalized treatment, which are distinct clinical questions. IoMT deployment is most defensible for remote screening when image quality is checked and positive or uncertain outputs trigger confirmatory examination [106].

Transformer-based cancer studies exploit long-range dependencies, but thyroid diagnosis illustrates the need to separate image, molecular, and tabular tasks. A method that succeeds in one modality does not provide evidence for the others merely because they share a disease label [107].

A conditional-GAN digital-twin system uses 5G-connected computation for surgical simulation. The generative model can synthesize or transform representations, but simulation realism and procedural safety require validation against physical or clinical outcomes; network integration alone cannot establish fidelity [108]. Multi-feature CNN fusion for EEG seizure prediction combines complementary representations within a consumer-IoT design. Fusion is valuable when each branch adds independent information, but it increases model size and leakage risk if correlated transformations of the same subject appear across splits [109]. CNN fusion for imagined movement in an IoMT brain–computer interface demonstrates that several representations can improve discriminative power. The result remains task-specific: controlled imagination classification does not directly translate to reliable assistive control under fatigue, motion, or long-term electrode drift [110].

A CNN plus gradient-boosting pipeline for driving-fatigue EEG combines learned and classical stages. Hybridization can improve accuracy while preserving a compact final classifier, but evaluation should include subject-independent testing because fatigue signatures are strongly individual [111]. A dual-input spatial-attention CNN embedded in a blockchain-secured medical-image system separates two concerns that should be evaluated separately: predictive validity and transaction integrity. Blockchain can protect provenance while leaving model bias, calibration, and adversarial image manipulation unresolved [112].

An elaborate chronic-kidney-disease pipeline combines temporal convolution, fuzzy cognitive maps, and ensemble optimization. Complexity may capture complementary structure, but every added component increases tuning freedom and makes ablation essential; without it, the source of any gain is unclear [113]. Ensemble monitoring systems reduce variance when component errors are diverse. They may also increase latency and memory, so robustness claims should include device measurements and failure correlation rather than accuracy alone [114].

Computational-intelligence reviews correctly position big-data analytics as a systems capability. Their breadth is useful for taxonomy, while specific deployment decisions still require task-level evidence about data, compute, and clinical action [115]. Imaging-focused IoMT surveys show rapid diffusion across brain, eye, and lung applications. Bibliometric or topical prevalence indicates research attention, not comparative effectiveness, and should not be used to infer which clinical application is most mature [116]. Predictive modeling in reproductive medicine highlights the cost of confounding, small cohorts, and changing treatment practice. These constraints make external and temporal validation particularly important before connected prediction is used to influence care [117].

Maternal–fetal risk prediction demonstrates the potential of combining imaging and sensing. Because stillbirth is rare, discrimination must be interpreted with calibration, precision, and decision thresholds; otherwise a numerically strong model may generate an unacceptable burden of false alarms [118].

### 6.4. Multimodal Fusion and the Training Challenges of Medicine

Multimodal learning is valuable when waveforms, images, laboratory measurements, and notes answer complementary parts of one clinical question. Fusion should preserve modality-specific missingness and timing rather than concatenate features indiscriminately. A strong multimodal model must also show that each modality adds value through ablation and that performance degrades safely when one stream is unavailable [119].

Remote-healthcare evidence consistently identifies scarce labels, class imbalance, and distribution shift as stronger deployment barriers than raw model capacity. These problems interact: oversampling can distort calibration, transfer learning can import bias, and personalization can overfit a small local history. Evaluation must therefore reproduce the intended patient, device, and site boundaries [120]. Transfer learning for early-autism detection shows how pretrained imaging representations can support small clinical datasets. The method reduces training demand, but its clinical meaning depends on whether pretraining and evaluation populations are independent and whether age, scanner, or site cues provide shortcuts [121]. Noise-resilient quantum machine learning is an emerging attempt to maintain analytics on imperfect quantum hardware. At present, its relevance to IoMT is exploratory: a useful comparison must include end-to-end runtime, encoding cost, hardware noise, and a matched classical baseline rather than accuracy alone [122].

Time-series anomaly-detection surveys provide the methodological bridge between continuous sensing and alerts. The key distinction is whether an anomaly represents sensor failure, behavioral novelty, or clinical deterioration; a detector trained without that distinction can be accurate statistically and useless operationally [123]. Digital-twin-centered predictive healthcare further increases the importance of shift because the model is expected to update as the person changes. Longitudinal adaptation requires versioned data, calibration monitoring, and a record of which model produced each recommendation [124].

The practical conclusion is that a successful IoMT model is defined less by architectural novelty than by how its training and validation confront scarcity, imbalance, missing modalities, and shift. Section 11 returns to the metrics and split rules required to test those properties.

### 6.5. When to Prefer Which, and the Hybrid Norm

The choice between classical and deep methods is governed by data volume, interpretability needs, and available compute, and in practice the two are combined. Table 8 distills the trade-offs. The dominant deployed pattern is a cascade in which a lightweight model screens continuously on-device and escalates only suspicious cases upward, mirroring the compute continuum of Section 4; Prabha et al. survey exactly such AIoT arrangements and their emerging technologies [125].

### 6.6. Making Deep Models Fit: Compression and TinyML

A deep model that cannot meet a device’s memory, latency, and energy budget is unusable at the point of care. TinyML unites pruning, quantization, distillation, compact architectures, and hardware-aware deployment. Compression should be judged on the target processor and medical task because nominal model size does not reveal end-to-end energy or accuracy loss [126].

Neuromorphic computing based on spiking neural networks offers a longer-term route to low-power processing of cardiac signals in wearable electronics. Its clinical relevance depends on demonstrating reliable inference, adaptation, and energy efficiency on physical devices rather than only in software simulations, so it remains an emerging hardware direction rather than a proven diagnostic solution [127]. Table 9 summarizes the principal techniques and their trade-offs.

## 7. Federated and Edge Intelligence for Privacy-Preserving Healthcare

**Figure 5:**
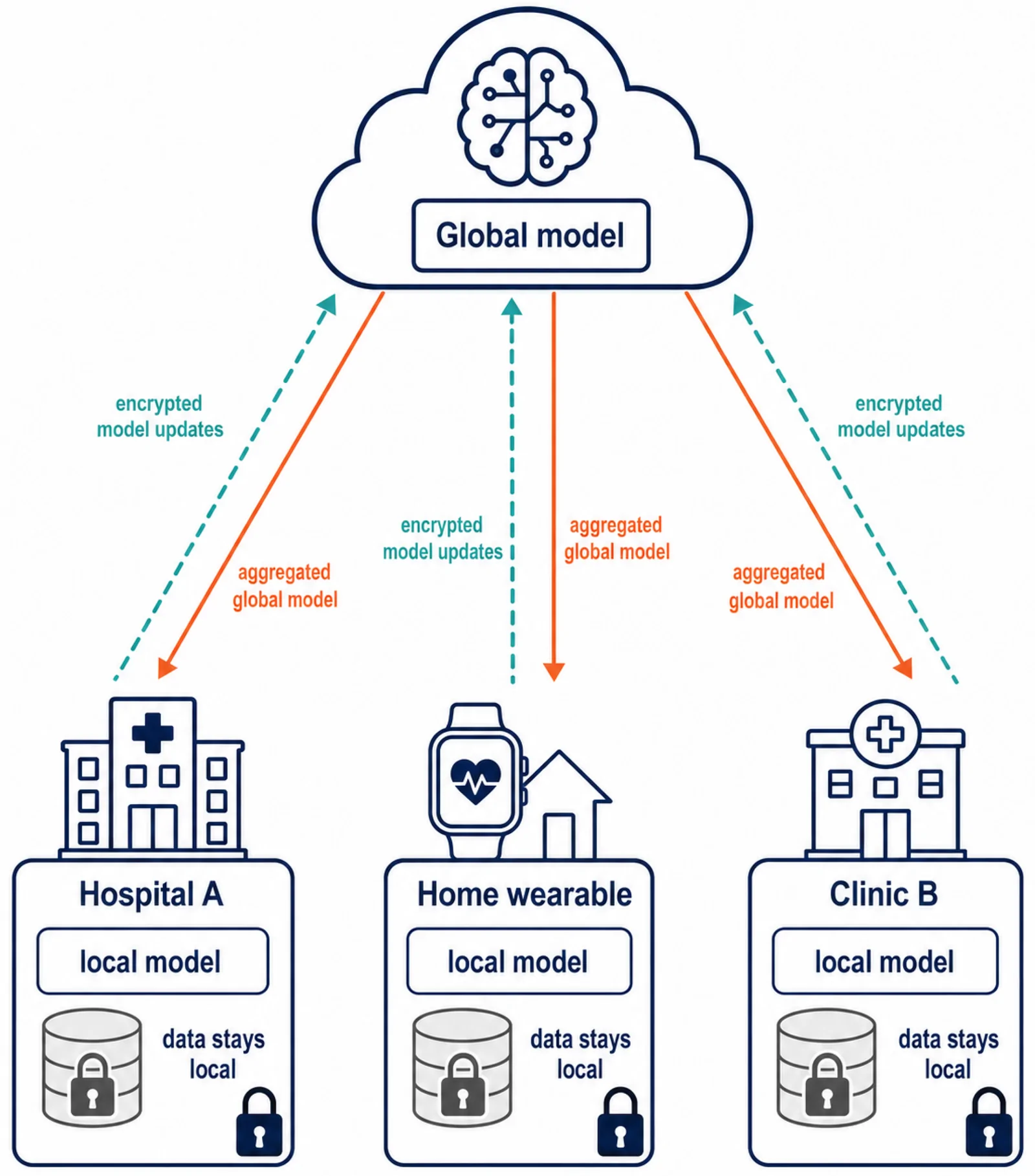
Federated learning in healthcare. Institutions and devices train locally and share only model updates with an aggregator; raw patient data never moves, aligning learning with privacy obligations.

Medical data is both the fuel of AI and among the most tightly protected categories of personal information, and this tension is the single most important force shaping how learning is organized in the IoMT. Federated learning (FL) resolves it in principle by training a shared model through the exchange of model updates rather than raw records, so that data never leaves its home. Figure 5 depicts the protocol, and this section examines why it fits medicine, the obstacles it faces, and the privacy machinery that hardens it.

### 7.1. Why Federated Learning Fits Medicine

Federated learning addresses two distinct problems: governance may prevent centralizing patient records, and any one site may lack representative cases. Medical taxonomies show that federation can support diagnosis, prognosis, imaging, and biosignal analysis, but they also make clear that keeping raw data local is not a complete privacy guarantee because gradients, participation, and model outputs can leak information [128].

Healthcare federation spans hospitals, research facilities, wearables, and gateways with very different compute and data distributions. A useful design must therefore specify the client unit, local task, aggregation rule, threat model, and what happens when a client is unavailable. Without those details, “federated” describes topology rather than an assurance property [129]. A data-centered view explains why federation is difficult in medicine. Sites differ in prevalence, acquisition devices, labels, missingness, and sample volume. More clients do not automatically create a more representative model; weighted averaging can instead privilege the largest or easiest sites [130].

Healthcare-5.0 surveys place FL within a human-centered, distributed service. Their useful contribution is a taxonomy of privacy, communication, and heterogeneity problems. Their broader claims about transformation remain conditional on demonstrating that a federated model improves performance or access relative to locally trained and centralized baselines [131]. An FL disease-diagnosis mechanism for IoMT evaluates privacy-preserving distributed training as part of the prediction pipeline. The key evidential question is whether the design reports per-client and global performance under non-IID data; a strong global average can conceal failure at a small hospital or subgroup [132]. Federated histopathology shows the paradigm’s value for images that cannot be freely pooled. Magnified views can be fused without sharing source slides, but patient-level separation remains essential because several patches from one tumor can otherwise leak across training and testing [133].

Federated ECG decision-making across edge, fog, and cloud links training topology to compute placement. Its benefit must be measured jointly in arrhythmia performance, communication rounds, energy, and latency; otherwise an accurate global model may still be unsuitable for continuous monitoring [134]. Comparisons between biosignal and EHR federation reveal that modality changes the statistical problem. Waveforms create high communication volume and device variation, whereas EHR data create semantic and missingness heterogeneity. One aggregation strategy should not be assumed optimal for both [135].

Federated reinforcement learning extends privacy-preserving training to sequential actions in consumer-centric IoMT. This raises the assurance burden because an unstable policy can affect future patient state; offline reward and convergence are not substitutes for constrained, clinically supervised evaluation [136]. General FL-scheme analyses supply a common vocabulary for centralized aggregation, decentralized exchange, and client selection. Medical use requires adding safety and accountability: the system must identify which model version acted, which clients contributed, and whether an update can be rolled back [137].

Multimodal federation compounds both modality missingness and site heterogeneity. A client may lack an entire signal type, so algorithms must distinguish an absent modality from a negative finding and avoid forcing incompatible feature spaces into a common update [138].

### 7.2. The Hard Problems: Heterogeneity, Communication, and Leakage

*The federated objective, formally.* Federated learning replaces the single dataset of (3) with *K* client datasets $D_{k}$ held at hospitals or devices. The global objective is a weighted sum of the local empirical risks $F_{k}\left(\theta \right)$,

(5) $$\begin{array}{cc} \min_{\theta }\;F\left(\theta \right) & =\sum_{k=1}^{K}\frac{n_{k}}{n}\;F_{k}\left(\theta \right),\\ F_{k}\left(\theta \right) & =\frac{1}{n_{k}}\sum_{j\in D_{k}}\ell \;\left(f_{\theta }\left(x_{j}\right),y_{j}\right), \end{array}$$

which the Federated Averaging algorithm optimizes by having each client take local gradient steps and the server average the resulting parameters $\theta^{t+1}=\sum_{k}\left(n_{k}/n\right)\;\theta_{k}^{t+1}$. Equation (5) is exact only when the clients are statistically identical; in medicine they are not.

The obstacles to FL are as real as its appeal. Statistical heterogeneity arises because each hospital sees a different case mix, so naive averaging converges poorly and personalization or adaptive aggregation is required. This can be made quantitative: the severity of the non-IID problem is captured by the divergence between each client’s gradient and the global gradient,

(6) $$\delta_{k}=∥\nabla F_{k}\left(\theta \right)-\nabla F\left(\theta \right)∥,$$

The raw value $\delta_{k}$ is scale dependent: it changes with loss scaling, model dimension, parameterization, batch construction, and local sample size. It should therefore not be compared across models without context. A dimensionless diagnostic can instead be reported as ${\tilde{\delta }}_{k}={∥\nabla F_{k}-\nabla F∥}_{2}/\left({∥\nabla F∥}_{2}+\epsilon \right)$, with ϵ>0 stated, or as a root-mean-square divergence normalized by parameter dimension. The aggregation weights $n_{k}/n$, the evaluation point θ, and the averaging interval should accompany either measure.

When divergence is large under a stated normalization—clients pulling in different directions—averaged updates may cancel and convergence can stall, motivating the adaptive-aggregation and personalization strategies of Table 10. System heterogeneity arises because devices differ in speed and availability, so the aggregator must tolerate stragglers. Communication cost is often binding because many rounds of update exchange can overwhelm limited uplinks. Local data are not the only attack target. Generative inversion attacks reconstruct private inputs from representations exchanged during collaborative inference, demonstrating that an architecture can satisfy “data stays local” while still exposing patient information. Evaluation must therefore use an explicit attacker with access consistent with the proposed protocol [139].

Federated-healthcare surveys synthesize privacy, non-IID data, communication, and personalization as one coupled design problem. Their evidence does not support choosing FL automatically; when governance permits secure centralization and sites are small, federation may add complexity without improving generalization [11]. An edge-assisted FL framework keeps local computation near the data and reduces dependence on a remote cloud. Its architectural benefit is plausible, but the deployment claim requires measurements of rounds, bytes, stragglers, and target-hardware energy in addition to model accuracy [140]. Table 10 contrasts the main coping strategies.

### 7.3. Hardening Federation: Privacy, Blockchain, and New Variants

Because FL reduces but does not eliminate privacy risk, it is routinely combined with additional protections. Differential privacy adds calibrated noise so that no individual’s contribution can be isolated. Formally, a randomized mechanism M is (ε,δ)-differentially private if, for any two datasets $D,D^{\prime }$ differing in a single record and any output set S,

(7) $$\mathrm{Pr}\left[M\left(D\right)\in S\right]\leq e^{\varepsilon }\;\mathrm{Pr}\left[M\left(D^{\prime }\right)\in S\right]+\delta ,$$

so that a smaller privacy budget ε forces the outputs on neighboring datasets to be more nearly indistinguishable—and therefore requires more noise, which lowers accuracy.

For an approximate-DP claim to be non-vacuous, δ must also be stated and chosen substantially below the inverse of the protected population size (a common convention is δ≪1/n), with the neighboring-record definition and composition across training rounds reported. Equation (7) formalizes both privacy-versus-accuracy and energy-versus-privacy tensions: stronger protection consumes utility and the mechanisms that enforce it consume device resources [141].

Local differential privacy protects records before updates leave a device, reducing trust in the aggregator. The price is noise applied before population aggregation, which can be severe for small clients or rare clinical classes. A reported privacy budget is meaningful only when paired with task performance and composition across all training rounds [141]. The same trade-off becomes operational in real-time federated-healthcare analyses that consider accuracy together with response requirements. The appropriate configuration is pathway-specific: an urgent alarm may tolerate less privacy-induced sensitivity loss than a population-level research model [142].

Blockchain-assisted federated learning can audit model-update exchange and reduce dependence on one aggregator. A healthcare-IoT implementation demonstrates secure transmission around a federated deep model, but ledger integrity does not ensure that local training data or updates are truthful [143]. Moving from model exchange to adversarial behavior, fraud-prevention designs combine blockchain and FL to identify malicious behavior in Healthcare 5.0 services. Their value depends on whether the threat model distinguishes billing or identity fraud from poisoning of the medical model; these require different labels and responses [144].

A secure Healthcare-5.0 platform entangles blockchain with federation to distribute trust. This can improve provenance while increasing latency, storage, and governance complexity, so comparisons should include a secure non-blockchain baseline rather than assume the ledger is necessary [145]. At the network layer, blockchain-backed federated intrusion detection shifts the learning target from disease to network behavior. It is valuable for protecting the IoMT service, but strong attack classification should not be interpreted as evidence that the clinical model remains safe after an intrusion [146]. Across these implementations, a systematic review of blockchain-secured medical FL finds recurring goals of auditability, decentralized trust, and privacy. It also exposes a maturity gap: many studies are simulations or prototypes and report inconsistent security and performance metrics, limiting comparative conclusions [147].

Broader privacy-preservation reviews position secure aggregation, differential privacy, encryption, and access control as complementary. No single mechanism covers confidentiality, integrity, availability, and authorized clinical use, so the protection stack must follow a stated threat model [148]. Where federation remains too demanding, split learning partitions a model between client and server and can keep raw input and complete model parameters apart. Applying it to large language models in smart healthcare may reduce local memory, but intermediate activations remain vulnerable and communication can exceed that of compact FL [149].

A more immediate efficiency route uses feature selection with a modified artificial-bee-colony method to reduce federated heart-disease input dimensionality. Any improvement must be assessed with selection confined to training folds, because selecting features before the patient split leaks information into evaluation [150].

Security-oriented surveys that integrate ML, FL, and explainability correctly connect prediction with trust. Their central implication is that a medically accurate model is not trustworthy if its data path, update mechanism, or explanation interface can be manipulated [151]. Federated emergency-medicine systems illustrate why timeliness and accountability matter together. Distributed learning may improve access to diverse cases, but the deployed decision path must identify uncertainty and preserve clinician authority for high-risk actions [152].

Federated and edge intelligence can reconcile bedside computation with data-governance constraints, but only when privacy, heterogeneity, communication, and clinical validity are measured together. Federation is an engineering arrangement, not evidence of privacy or benefit by itself.

## 8. AI-Enabled Diagnostic Pathways in the IoMT

**Figure 6:**
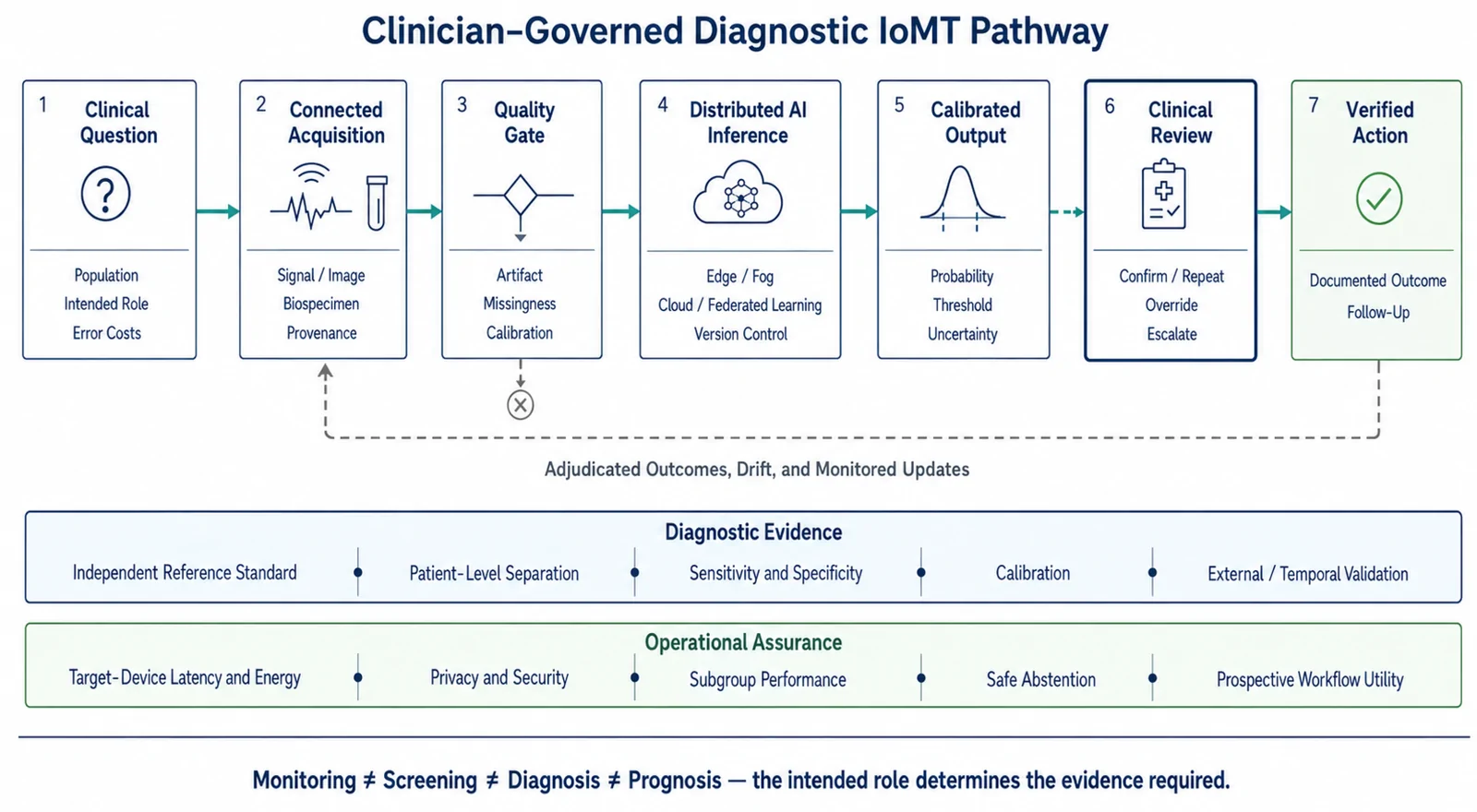
Clinician-governed diagnostic pathway for the IoMT. A model output becomes clinically interpretable only when the target population, reference standard, quality gate, threshold, uncertainty policy, and confirmatory action are specified. Feedback contains adjudicated outcomes and device or site drift rather than unverified model predictions.

The preceding sections describe the components of connected intelligence; this section assembles them into a diagnostic pathway. Figure 6 begins with a clinical question rather than a device. The question determines the target population and reference standard; these determine what should be sensed and how labels are assigned. Quality control then decides whether a measurement is interpretable before any model is allowed to classify it. The output is calibrated and compared with a prespecified decision threshold, after which uncertain, poor-quality, or high-risk cases are referred for repeat or confirmatory assessment. This order prevents a common inversion in which an available dataset defines the clinical task after model development.

### 8.1. From a Measurement to a Diagnostic Claim

The first boundary is between a valid measurement and a valid label. A sensor may measure an electrical potential, optical response, acceleration, image intensity, or molecular binding event; none of these is a diagnosis without a defined mapping to disease. In electrocardiography, the reference may be expert interpretation of a standard multi-lead recording; in seizure studies it may be neurologist-adjudicated video EEG; in imaging it may be pathology, follow-up, or expert consensus; and in point-of-care testing it may be a laboratory assay. The reference need not be perfect, but its construction and independence from the index model must be reported because noisy or circular labels cap the validity of every subsequent score [5,153].

The second boundary is between technical detection and the intended clinical use. A highly sensitive wearable rule may be appropriate for screening if positive cases receive confirmatory assessment, even when its specificity is inadequate for diagnosis. Conversely, a model optimized for balanced accuracy on a case–control dataset may be poorly calibrated for a low-prevalence screening population. Threshold selection must therefore be tied to prevalence, the harm of missed disease, the burden of false referrals, and the availability of confirmation. The same classifier can be defensible as a triage aid and indefensible as an autonomous diagnostic device.

Data separation is part of this boundary. Multiple windows, images, or visits from one patient must remain within one partition; otherwise identity and acquisition features can leak from training into testing. Site-held-out and later-time evaluation answer further questions: whether performance transports to new equipment and practice patterns, and whether it survives temporal change. High internal discrimination is useful for model development, but it does not substitute for external validation, calibration, or prospective evaluation of the complete pathway [6,154].

### 8.2. Connected Physiological Signals: ECG and EEG

Physiological waveforms are a natural IoMT use case because events may be intermittent and clinically important. For ECG, connected acquisition increases the probability of recording transient rhythm abnormalities, while multilead analysis retains diagnostic relationships that single-channel wearables may lose. TGLLNet illustrates this distinction by learning task-guided relationships among twelve ECG leads for multilabel classification rather than treating leads as independent or merely adjacent channels [155]. Its methodological contribution is relevant to diagnostic design, but deployment evidence must still establish how performance changes when fewer wearable leads, different electrode positions, motion artifact, or a new device alter those relationships.

Distributed training can reduce the need to centralize ECG data, yet a federation is clinically useful only if it preserves minority-class sensitivity at each participating site. A laboratory deployment on heterogeneous physical IoT devices demonstrates that ECG-based clinical decision support can be trained federatively outside a purely simulated client environment [156]. That moves systems evidence forward, but it does not by itself prove improved diagnosis: per-site calibration, patient-level external testing, communication failures, and clinician response to the output remain separate endpoints.

EEG-based seizure recognition exposes a different error asymmetry. A missed event can delay intervention, while repeated false alarms can create alarm fatigue and unnecessary escalation. SMARTSeiz uses attention-based deep learning to address nonstationary EEG and variable seizure patterns on IoT healthcare devices [157]; weighted visibility graphs offer a complementary route by encoding nonlinear EEG structure within a device–fog–cloud framework [158]. These methods should not be compared solely by average accuracy because their representations, datasets, and operating points differ. A useful comparison reports event-level sensitivity, false alarms per hour, detection delay, patient-held-out performance, and energy on the intended recorder.

An additional challenge is diagnostic specificity. VPSI 2.0 seeks real-time separation of psychogenic nonepileptic seizures from generalized tonic–clonic seizures using a vibration-based IoMT protocol [159]. The clinical question is important because the conditions require different management, but vibration is an indirect measurement of cortical pathology. Such a system is better positioned as an escalation or triage signal until it is compared prospectively with video EEG and neurological assessment across representative mimics.

### 8.3. Connected Imaging and Computer-Aided Diagnosis

IoMT imaging extends beyond transmitting images to a server. A connected diagnostic service must preserve acquisition protocol, image identity, preprocessing history, and model version, and must return an output that a radiologist or other specialist can relate to the source image. Medical image segmentation illustrates why this matters: MSCL-Net targets multiorgan abdominal segmentation where organ size and ambiguous boundaries challenge multiscale representation [160]. Accurate segmentation can support measurement and treatment planning, but segmentation overlap is an intermediate endpoint; diagnostic value depends on whether the contours improve lesion detection, staging, planning, or reporting.

For lung abnormalities, an explainable IoMT framework can classify medical data into benign and malignant groups and expose features used by the model [161]. The explanation does not validate the diagnosis by itself. It should be tested for localization fidelity, stability under clinically irrelevant perturbations, and usefulness to the intended reader, while the classifier requires independent confirmation and an evaluation spectrum that includes benign mimics. A more recent interpretable medical-image framework combines deep networks, symbolic reasoning, confidence-weighted attention, and adversarial observability, with evaluations on CheXpert, the RSNA pneumonia challenge, and NIH chest radiographs [162]. Its joint treatment of explanation and attack robustness is valuable for connected systems, but multi-dataset evaluation is not automatically external clinical validation if development choices were informed by all datasets or if patient care was not prospectively affected.

Leukemia classification demonstrates both the reach and the boundary of connected microscopy. An IoMT-enabled feature-fusion framework classifies acute lymphoblastic leukemia from blood-cell imagery, potentially supporting remote access to computational review [163]. Image classification cannot replace the full diagnostic work-up, which may include blood counts, immunophenotyping, bone-marrow examination, cytogenetics, and molecular testing. The defensible IoMT role is therefore preliminary classification or prioritization unless the evaluation explicitly establishes equivalence to the clinical diagnostic standard.

### 8.4. Biosensors and Decentralized Point-of-Care Diagnosis

Point-of-care sensors make the diagnostic IoMT materially broader than wearable telemetry. A connected assay couples biological recognition and transduction to identity, quality control, interpretation, and reporting. For leukemia, reviewed platforms include electrochemical, piezoelectric, surface-plasmon-resonance, colorimetric, lateral-flow, microfluidic, and smartphone-connected approaches targeting fusion genes, surface antigens, exosomes, and circulating tumor cells [8]. The network can support traceability and remote interpretation, but analytical sensitivity, selectivity, limit of detection, interference, specimen preparation, and agreement with the central laboratory must be established before connectivity has clinical meaning.

In infectious disease, molecular assays and AI-based computer-aided detection solve different parts of the problem. RT-PCR, antigen–antibody, and CRISPR biosensors seek evidence of a pathogen or immune response, whereas image or symptom models infer disease from associated patterns [7]. Combining them may improve triage, but a model output should not be reported as equivalent to pathogen detection. The most useful connected pathway records specimen time and quality, assay lot and calibration, patient identity, model version, and confirmatory result so that discordant cases can be investigated.

Decentralized testing can also move care without moving the patient. In maternity care, AI, IoMT, and point-of-care testing have been proposed to extend prenatal assessment and support an unevenly distributed workforce [164]. The diagnostic benefit depends on more than device availability: escalation thresholds, gestational-age-specific reference ranges, repeat testing, referral capacity, and equity of access determine whether a remote abnormal result changes outcomes. This illustrates a general principle—decentralization increases access only when the confirmation and treatment pathway is decentralized or reliably connected as well.

### 8.5. Calibration, Uncertainty, and the Referral Decision

A diagnostic IoMT system should be permitted to abstain. Forced classification turns missing leads, motion-corrupted signals, out-of-distribution images, and low-confidence predictions into apparently definite outputs. A quality gate can reject uninterpretable measurements, while an uncertainty gate can refer cases near the decision boundary or unlike training data. These gates alter throughput and referral burden, so coverage—the proportion of cases receiving an automated output—must be reported alongside performance on the retained cases.

Calibration connects probability to action. If 20 of 100 patients assigned a risk of 0.2 have the outcome, the model is calibrated at that range even if its ranking is imperfect; a highly discriminative but overconfident model may be more dangerous when its probability triggers discharge or reassurance. Calibration should be assessed overall and within clinically important subgroups, repeated after compression or site transfer, and monitored after deployment. Decision-curve analysis can complement sensitivity and specificity by examining the net consequence of thresholds, but it remains dependent on plausible clinical preferences and cannot replace a prospective workflow study.

Table 11 summarizes how evidence requirements change by modality. The unifying endpoint is not a universal accuracy score: it is whether the measurement, model, and referral policy together answer a specified clinical question with known uncertainty.

## 9. Diagnostic and Prognostic Application Domains

**Figure 7:**
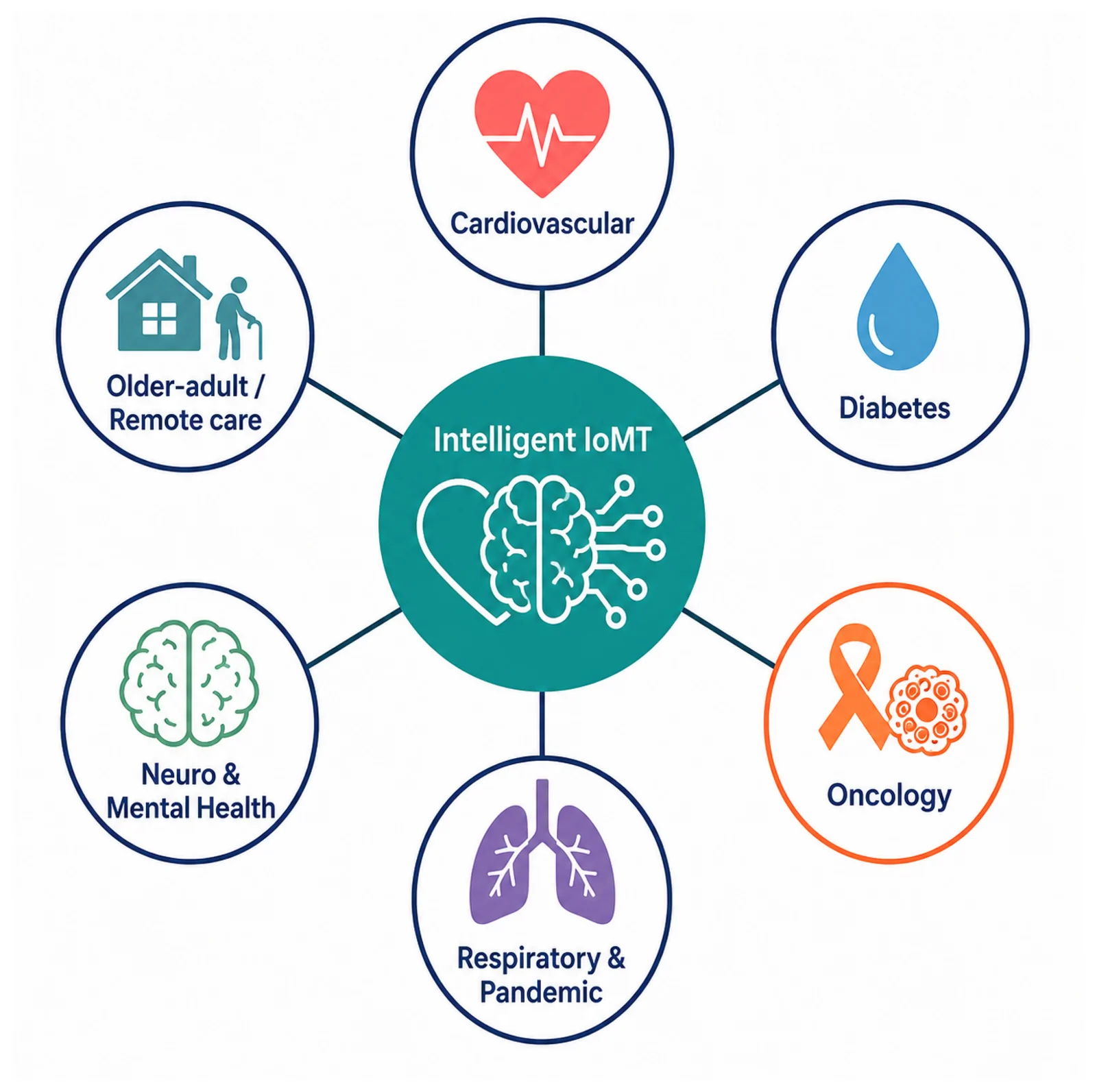
Diagnostic and prognostic application domains of the IoMT. The same sensor may support monitoring, screening, diagnosis, or prognosis; the clinical role is determined by the target condition, reference standard, threshold, and downstream action.

The diagnostic pathway takes a different form in each clinical domain. Cardiovascular and neurological systems emphasize intermittent waveform events; diabetes combines screening with longitudinal control; oncology joins imaging, pathology, and molecular confirmation; respiratory and infectious-disease pathways combine physiological monitoring with pathogen or imaging evidence; and older-adult systems are more often early-warning and functional-risk services than disease-diagnostic tools. The following analysis therefore identifies the defensible diagnostic role before comparing algorithms. Figure 7 and Table 12 summarize these domain-specific mappings.

### 9.1. Cardiovascular Screening, Rhythm Diagnosis, and Risk

Cardiovascular care is a natural IoMT use case because ECG and PPG can capture episodic abnormalities outside a clinic. Cardiology-focused reviews find plausible roles in screening, risk estimation, and workflow support, while also warning that algorithmic performance and clinical effectiveness remain different evidence levels [166].

An IoT-and-ML remote cardiac platform aims to move detection earlier and lower monitoring cost. Its practical value depends on acquisition continuity, alert latency, and false-alarm burden; a prototype classification result alone cannot demonstrate those service-level benefits [167]. Home cardiovascular monitoring increases observation time but transfers measurement responsibility to an uncontrolled environment. A home system must therefore demonstrate placement guidance, missing-data handling, escalation, and recovery after connectivity loss in addition to vital-sign acquisition [168]. ML-powered heart-disease identification shows how IoMT measurements can feed a risk classifier. Unless evaluation uses independent patients and measurements produced by the intended device, the result supports prediction on a dataset rather than the full connected system [169].

Integrated heart-disease prediction combines several processing stages to improve discrimination. The scientific value of integration should be demonstrated through ablation and external testing; otherwise additional stages may increase tuning freedom without improving transportability [170]. A recursion-enhanced random forest with an improved linear component preserves some of the efficiency and interpretability of classical learning. Its IoMT relevance is strongest when the complete preprocessing and inference path is tested on constrained hardware rather than inferred from algorithm complexity [171].

Cloud XGBoost and bidirectional-LSTM models for hypertension-related risk combine tabular and temporal analysis. Cloud execution provides capacity, but risk estimates still require calibration in the intended population and a defined response when the network is unavailable [172]. Adaptive deep networks can model nonlinear heart-disease patterns within connected monitoring. Adaptation becomes clinically meaningful only when update data, versioning, and rollback are controlled; an unmonitored adaptive model can drift after deployment [173]. An IoT monitoring system using deep learning can connect streaming variables to heart-disease prediction. Retrospective forecasting does not establish that an alert precedes deterioration early enough to alter management, so temporal and prospective validation remain essential [174].

Physics-guided cardiovascular learning constrains prediction with domain structure and can reduce dependence on large labeled datasets. Its advantage should appear under shift or sparse data, not only as a marginal accuracy gain on the same benchmark [175]. Reviews of IoT-based cardiac AI show that tasks range from beat classification to patient-level risk prediction. These outputs are not interchangeable: beat accuracy may support rhythm analysis but cannot be compared directly with prognosis or event prediction [176].

Broader cardiology syntheses demonstrate the reach of connected monitoring while identifying interoperability, trust, and validation as continuing limits. Their evidence supports the importance of the domain, not a claim that one model or sensor is ready for universal use [21]. ML-supported cardiac rehabilitation changes the target from diagnosis to adherence and functional recovery. Evaluation should therefore measure completed activity, safety events, and functional outcomes rather than reuse diagnostic accuracy metrics [177].

AI for cardiopulmonary resuscitation spans prediction, event recognition, and guidance. Because these tasks occur under extreme time pressure, prospective workflow and human-factors testing are more important than small retrospective gains [178]. An Efficient-Net cardiac-arrest predictor coupled to a digital twin illustrates patient-specific modeling. The twin label is justified only if the model is continuously synchronized and its counterfactual or temporal predictions are validated; otherwise it remains a connected risk model [179]. Blockchain-supported heart monitoring can strengthen provenance and access control around physiological data. It does not validate the sensing or medical decision rule, and ledger overhead must be included in latency and energy measurements [180].

### 9.2. Diabetes Screening, Glucose Forecasting, and Metabolic Risk

Diabetes generates both long-term risk data and high-frequency glucose trajectories, which support different AI tasks. Deep models in IoT sensing environments can classify diabetes risk, but this should not be confused with forecasting near-term glucose excursions or recommending therapy [181].

A home-centric urine-based IoT system offers a less conventional route to diabetes prediction. Its usefulness depends on analytical repeatability and confounders in urine measurement; remote connectivity does not convert a noisy proxy into a validated diagnostic marker [182]. Bayesian optimization of XGBoost addresses model tuning for complex, imbalanced diabetes data. Performance should be reported with precision, recall, calibration, and patient-level splits because high accuracy can be obtained by favoring the majority class [183].

Surveys of diabetes detection reveal extensive reuse of standard datasets and heterogeneous preprocessing. Their main implication is that small differences in reported accuracy rarely identify a clinically superior model without external or temporal validation [184]. Digital-diabetes-care reviews broaden the pathway from prediction to monitoring, self-management, and treatment support. These functions require different reference standards and outcomes, so they should not be pooled into one notion of “AI performance” [185]. An automated ML-operations pipeline combines IoT, edge processing, privacy, and blockchain for diabetes prediction. End-to-end automation can improve traceability, but it also requires controlled model updates and monitoring so that a new version does not silently change clinical behavior [186].

Information-management pathways can improve identification and follow-up by connecting glucose data with care processes. Their effect should be evaluated through completed testing, diagnosis, and treatment actions rather than inferred from data availability [187]. Work on insulin-therapy prescribing addresses the treatment pathway rather than sensor prediction. Because prescribing is causal and safety-critical, observational associations in connected records cannot by themselves establish which therapy should be chosen [188].

A lightweight language-model anomaly detector for implantable diabetes devices shifts the target to device security. Detecting abnormal communications may protect therapy, but it must be distinguished from detecting abnormal glucose or recommending insulin; each needs separate labels and safety tests [189].

### 9.3. Oncological Imaging, Pathology, and Molecular Detection

In oncology, “detection” can mean screening an eligible population, classifying a known lesion, segmenting a tumor, or identifying a molecular marker. IoT-based breast-cancer reviews often combine these tasks, so their reported methods must be interpreted against the exact input and reference label rather than the disease name alone [190].

Comparisons of AI techniques for breast images are useful when the modality, patient split, lesion spectrum, and metric are aligned. A ranking across mammography, ultrasound, histopathology, and thermography would otherwise compare different clinical questions [191]. An IoMT-embedded CNN for invasive ductal carcinoma classifies histopathology rather than performing population screening. Its connected architecture can support remote pathology, but clinical evidence requires independent slides and patients, stain variation, and pathologist comparison [192]. Multimodal oral-cancer AI can combine images, clinical findings, and molecular or behavioral data. Fusion is most credible when each modality’s added value is quantified and missing inputs do not force an unsupported prediction [193].

Kidney-tumor surveys cover detection, segmentation, classification, and prognosis across imaging tasks. These outputs should be separated because a high segmentation Dice score does not establish correct malignancy classification or treatment benefit [194]. A hybrid white-blood-cell cancer detector with an IoT alert mechanism joins image analysis to notification. The alert is clinically meaningful only if the classification label has an appropriate laboratory reference and the workflow routes uncertain cases for confirmation [195].

Machine-learning lung-cancer detection over medical-IoT devices demonstrates distributed access to prediction. It does not by itself establish early detection because evaluation must begin with the population eligible for screening rather than a balanced set of known cases and controls [196]. Cancer care in patients with multimorbidity exposes the limits of single-disease optimization. An accurate cancer model may recommend an action incompatible with cardiovascular, renal, or functional constraints, so connected decision support must represent competing risks and clinician judgment [197].

### 9.4. Respiratory and Infectious-Disease Detection

COVID-19 accelerated remote monitoring but also produced many studies on rapidly assembled datasets. Imaging reviews show high retrospective performance alongside dataset duplication, shortcut learning, and weak external validation; the enduring lesson is about validation under shift rather than a specific pandemic classifier [198].

AI contributed to imaging, forecasting, triage, and public-health surveillance during the pandemic. These tasks operate at different scales and should not be collapsed into a single claim that AI “contained” disease [199]. CronaSona combines detection, forecasting, and treatment advice in one ecosystem. Integration is ambitious, but every output requires its own reference standard and safety boundary; strength in one module cannot validate the others [200].

ML–AI–IoT pandemic pipelines demonstrate how sensing, communication, and analytics can be organized quickly. Their reuse beyond COVID-19 depends on whether components are disease-specific or represent general monitoring and escalation functions [201]. Internet-of-intelligent-things proposals for limiting spread emphasize distributed sensing and response. Their population-level surveillance benefits must be balanced against consent, proportionality, false positives, and the difficulty of linking an automated alert to effective action [202].

Smart-healthcare solutions for continuous monitoring and stratification show how remote observations can reduce unnecessary contact. Stratification remains a clinical decision and needs calibrated risk, clear thresholds, and confirmation rather than an unqualified class label [203]. Digital-imaging reviews document the speed with which AI models were adapted during COVID-19. They also demonstrate that emergency development can outpace evidence quality, creating a need for locked external testing before clinical claims [204]. Respiratory-sound classification offers a low-cost path to airway monitoring. Real-time feasibility is valuable, but cough and breath acoustics vary with environment, microphone, age, and comorbidity, so device- and site-independent testing is essential [205].

Combining physiological and environmental sensors can improve early respiratory-risk context. Environmental correlation is not necessarily causal, and models must avoid using location or season as shortcuts that fail after deployment [206]. Smartphone photonic sensing broadens remote access by using widely available hardware. Its clinical value depends on optical calibration across phone models, ambient conditions, and user technique rather than scalability alone [207].

Under-mattress ballistocardiography enables contactless monitoring and a multitask model can estimate several vital signs from one sensor. Performance must be tested across mattresses, posture, body habitus, and multiple occupants before home deployment [208]. Thermal smart spectacles and capsule networks illustrate wearable symptom screening. Temperature patterns are nonspecific, so the system is better interpreted as a triage aid whose positives require confirmation than as a diagnostic device [209].

### 9.5. Neurological Event Detection and Mental-Health Assessment

Neurological and mental-health applications use EEG, actigraphy, speech, physiology, and behavior, but their labels are often subjective or temporally unstable. A seizure-warning system based on multidimensional attention entropy targets an actionable pre-event window; evaluation should therefore report warning time, false alarms per hour, and patient-level sensitivity rather than accuracy alone [210].

IoMT seizure-detection surveys show rapid algorithmic progress and recurring dependence on a few public EEG datasets. The main gap is generalization across subjects, electrodes, and recording conditions, which limits claims of continuous use [211]. Explainable seizure detection can reveal which EEG regions or features influenced a prediction. Explanations are clinically useful only if they are stable, faithful to the model, and evaluated with neurologists rather than presented as reassuring visualizations [212].

Intelligent-IoMT reviews for depression propose passive or minimally supervised monitoring. Because depression is not directly observable from one signal, these systems should support assessment and follow-up rather than claim diagnosis without a validated clinical reference [213]. Video emotion recognition in social IoT can capture behavioral cues relevant to mental health. Emotion classes are context- and culture-dependent, so performance on posed or curated videos does not establish clinical state recognition [214].

Real-time modeling for women with menstrual disorders connects physiological context with mental-health observations. Survey-derived or self-reported labels require careful temporal alignment and confounder control before predictive associations are interpreted clinically [215]. Online mental-health AI reviews identify adherence and dropout as central outcomes. A model cannot help a user who disengages, which makes usability, escalation, and harm monitoring as important as prediction [216]. Cross-dataset evaluation of HRV stress models directly tests transportability. Performance loss across datasets is scientifically more informative than a high within-dataset score because it reveals sensitivity to sensor, protocol, and population [217].

Fusing galvanic skin response with ECG can improve stress classification by combining autonomic signals. Both respond to exercise, illness, and environment, so multimodal agreement does not by itself make the label specific to psychological stress [218]. An intelligent device that monitors food intake and stress illustrates long-duration behavioral sensing. Its value lies in linking patterns over time, but privacy and user burden must be included because continuous behavioral observation can itself alter adherence [219].

AI-supported soft robotics for Parkinson’s disease moves from observation to physical intervention. This raises the evidence level from classification to safety, controllability, and functional improvement in the intended patient population [220]. IoMT-supported stroke rehabilitation combines sensors, feedback, and remote supervision. Appropriate outcomes include functional recovery, adherence, and clinician workload; movement-classification accuracy is only an intermediate measure [221]. A secure sleep-health framework combines sensing and classification with network protection. Sleep-state performance and intrusion resistance answer different questions and should be reported independently before the complete system is called trustworthy [222].

### 9.6. Older-Adult Screening, Functional Risk, and Remote Monitoring

Assisted living and remote monitoring place a premium on reliability because false alarms create caregiver burden while missed events can delay care. An edge-AI architecture for early detection reduces network dependence, but “early” must be defined relative to an actionable event and measured prospectively [223].

An AI-driven telemedicine framework combining IoMT, cloud resources, and wireless sensing demonstrates service integration. Its effectiveness depends on whether alerts are received and acted on, not simply whether data reach the cloud [224]. Reviews of deep-learning monitoring platforms show growing commercial interest. Product availability is not equivalent to clinical validation, and proprietary data can make independent comparison more difficult [225].

Big-data monitoring syntheses trace the path from sensors to insights. Their key systems lesson is that volume and continuity increase the need for data-quality checks and summarization so clinicians are not presented with an unfiltered stream [226]. Successful-ageing reviews identify privacy, interoperability, accessibility, and adoption as core barriers. These are outcome determinants: a technically accurate system that an older adult cannot use or afford has no real-world sensitivity [227].

Non-wearable anomaly detection can infer deviations from routine without requiring adherence to a device. Its main risks are ambiguous attribution, changing household routines, and treating novelty as illness; caregiver feedback is therefore needed to interpret alerts [228]. AIoT human-activity-recognition surveys show applications across safety, rehabilitation, and daily-living assessment. Activity labels and required granularity differ by application, so benchmark accuracy cannot be transferred directly from fitness recognition to fall prevention [229]. Optimized ML for activity recognition in cyber-physical systems demonstrates algorithmic feasibility. Optimization must remain inside the training procedure and evaluation should be subject-independent to avoid learning an individual’s motion signature [230].

Sensing-and-computing reviews for activity analysis reveal trade-offs among wearables, cameras, radio sensing, and ambient devices. Privacy, occlusion, placement, and multi-person ambiguity differ substantially, making sensor choice a clinical and environmental decision [231]. Wearable HAR reviews emphasize accelerometers and inertial sensors as scalable inputs. Long-term performance still depends on placement consistency, charging, and whether the user actually wears the device during high-risk periods [232].

Accelerometer-based edge fall detection shows why local inference is attractive for urgent events. A useful evaluation must include real or realistic falls, activities that mimic falls, on-device latency, and false alarms over long monitoring periods [233]. Deep-learning reviews of older-adult behavior confirm that falls are only one target among wandering, inactivity, and changes in routine. Combining them in one model requires separate thresholds and response pathways because their urgency differs [234]. A BERT-based medical chatbot can reduce communication friction and provide accessible interaction. It must not be treated as a diagnostic authority unless retrieval, uncertainty, escalation, and harmful-response controls are explicitly validated [235].

Telemedicine for fishermen at sea illustrates the value of connected care under geographic isolation. The architecture must tolerate intermittent links and define which functions remain available offline, making resilience more important than average throughput [236]. Scoping reviews of intelligent cyber-physical healthcare systems connect sensing with actuation and workflow. Their broad taxonomy is useful, but the evidence level must rise sharply when software can change therapy rather than merely observe [237].

Remote perioperative monitoring extends observation beyond the operating room and may detect deterioration earlier. Because postoperative events are uncommon, studies should report alert burden, escalation success, and unplanned-care outcomes rather than accuracy alone [238]. AI in dialysis and kidney transplantation demonstrates a domain with repeated measurements and high-risk decisions. Models should account for longitudinal dependence and treatment changes; randomly splitting repeated visits can otherwise produce optimistic performance [239].

### 9.7. The Validation Gap: From Accuracy to Clinical Deployment

Strong retrospective accuracy and routine diagnostic use are separated by several evidence steps. The intended use must first identify the population, setting, operator, input, output, and consequence of error. Evaluation then requires patient-level separation and an independent reference standard, followed by locked external or temporal validation. A deployable connected test must additionally demonstrate robustness to device and site shift, acceptable uninterpretable-test and false-alert rates, and a workflow in which an accountable recipient can confirm, override, or escalate the output. Digital-health innovation reviews show that technical promise becomes clinical value only after this organizational and evidential integration [6].

Diagnostic metrics should be reported at the chosen threshold. Sensitivity and specificity describe complementary error rates; positive and negative predictive values reveal the effect of prevalence; likelihood ratios help relate a result to pre-test probability; and calibration assesses whether predicted risks have quantitative meaning. Imaging segmentation additionally needs task-specific geometric metrics, while continuous event detection needs event sensitivity, false alarms per patient-time, and detection delay. Confidence intervals and subgroup results are required because a single point estimate can conceal clinically important uncertainty or unequal failure.

The engineering trade-offs interact directly with these metrics. Compression can make a model fit a wearable while weakening minority-class sensitivity; differential privacy can change calibration; and edge placement can improve latency while complicating update control and audit. Explainability can support review, but cannot replace prospective diagnostic performance or human-factors evaluation [154]. The benchmark winner is therefore not necessarily the deployable model. Clinical selection favors a pathway whose threshold, uncertainty, provenance, security, reference standard, and failure behavior remain defensible under regulated real-world use [240].

## 10. Trustworthy Diagnostic IoMT: Security, Privacy, and Explainability

**Figure 8:**
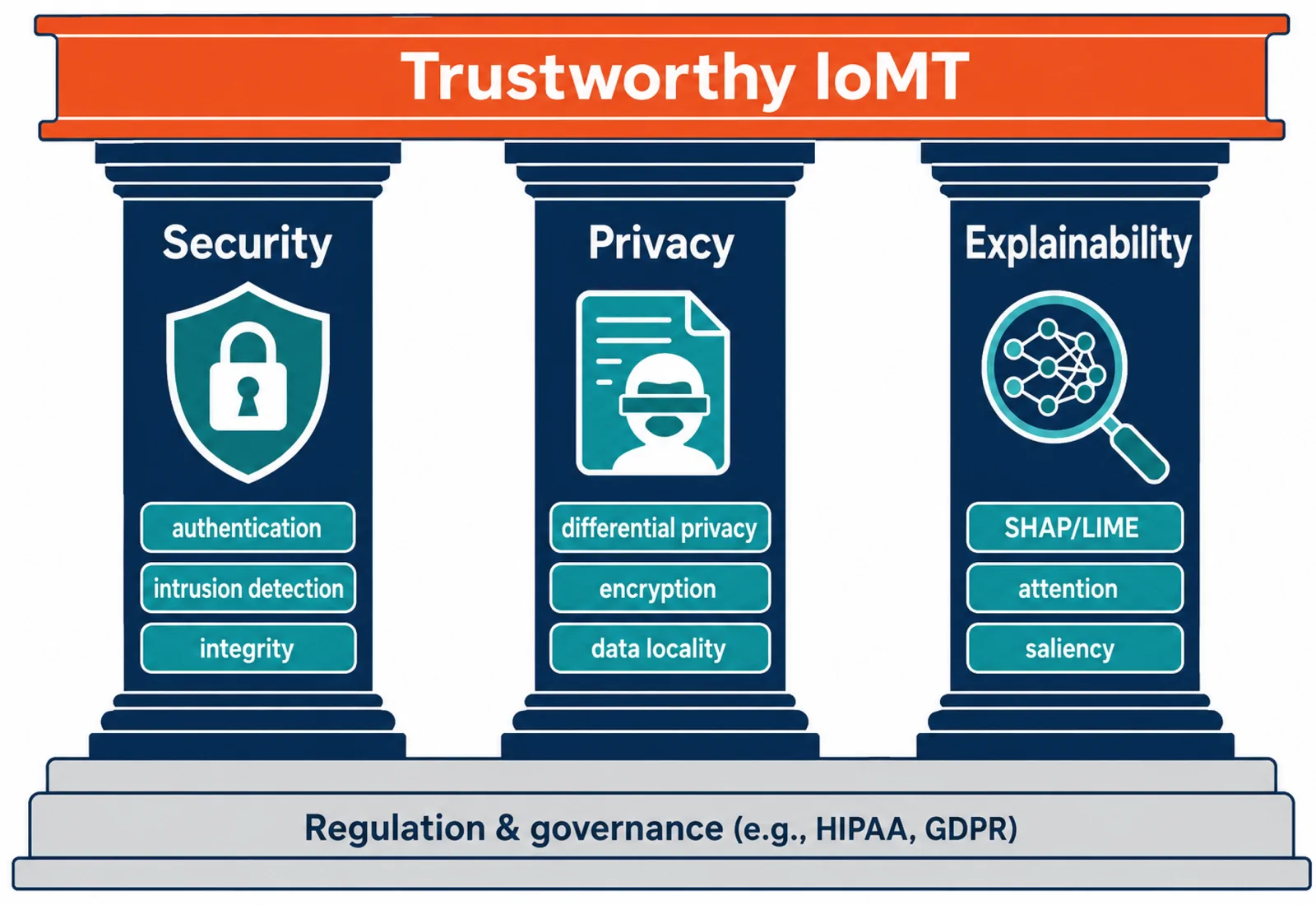
The three pillars of a trustworthy IoMT. Security, privacy, and explainability rest on a foundation of regulation and governance; all four are prerequisites for clinical deployment of learning-based systems.

The diagnostic applications above show that model error is only one route to harm. A compromised sensor can alter the index measurement, a manipulated update can change sensitivity, a privacy breach can expose disease status, and a misleading explanation can encourage inappropriate reassurance. Trustworthiness therefore forms part of diagnostic validity and follows the signal across the whole pathway. Figure 8 organizes this requirement into three interlocking pillars.

### 10.1. The Attack Surface and Its Defenses

The IoMT attack surface spans low-power endpoints, gateways, cloud services, clinical accounts, update channels, and models. Security reviews show that AI can improve detection while creating new assets and attack paths of its own. A defensible architecture must therefore protect availability and integrity of the medical function, not only confidentiality of stored data [14].

Adversarial-machine-learning surveys organize model-level threats into evasion, poisoning, extraction, inversion, and backdoors. Their practical importance varies with attacker access, so every defense should state whether the adversary can modify training data, device inputs, updates, or only network traffic [241]. AIoT-specific threat analyses demonstrate how evasion and model manipulation interact with heterogeneous edge deployment. A robust classifier alone is insufficient if firmware, sensor identity, or the update channel can be compromised [242].

Stacking, bagging, and boosting can improve intrusion detection by combining classifiers with different error patterns. Ensemble gains should be evaluated under temporal or device-held-out splits because random traffic splits often place nearly identical flows in training and testing [243]. RCLNet uses anomaly-oriented learning to model complex IoMT traffic beyond fixed signatures. Anomaly detection can identify novel behavior, but it also produces false positives when legitimate software or clinical workflows change; deployment therefore needs feedback and retraining controls [244]. Benchmarking on a modern IoMT dataset exposes severe class imbalance among attack types. Macro-averaged recall and per-attack detection are more informative than overall accuracy, particularly when rare attacks have the greatest safety consequence [245].

Optimized feature selection reduces IDS computation and may fit constrained gateways. Selection must occur within training folds, and the retained features must remain observable after protocol or firmware changes [246]. Comprehensive feature-selection studies similarly show that a smaller traffic representation can preserve detection. Their real deployment value depends on measuring extraction cost as well as classifier cost, because expensive features can erase the benefit of a compact model [247].

Graph neural networks represent relations among devices and flows rather than classifying each record independently. This can expose coordinated behavior, but graph construction and identity assumptions become part of the attack surface and must be tested under missing or spoofed nodes [248]. A top-down ML architecture provides a general hierarchy for identifying and classifying IoT cyberattacks. Its concepts transfer to IoMT only after medical protocols, constrained devices, and safety-critical response requirements are represented in the evaluation [249].

Blockchain-based anomaly detection can preserve event provenance while a deep network classifies traffic. Ledger integrity is valuable for audit, but it neither guarantees correct detector outputs nor prevents denial of service, and its overhead must be measured [250]. Reviews combining AI, blockchain, and IoMT cybersecurity show that these technologies solve different parts of the assurance case. AI detects patterns, blockchain records transactions, and conventional controls enforce identity and access; none replaces the others [251]. A chaotic-encryption framework for cognitive-healthcare IoT targets confidentiality with a distributed learning component. Cryptographic strength must be evaluated independently from prediction accuracy, and custom encryption should be compared with established lightweight standards [252].

Federated intrusion-detection surveys show how several organizations can learn without pooling raw traffic. Federation introduces poisoning, non-IID clients, and update leakage, so it redistributes trust rather than eliminating it [253].

### 10.2. Privacy and Explainability

Privacy concerns what can be inferred from measurements, metadata, updates, and model outputs, not merely whether transport is encrypted. Dermatological-image processing illustrates the end-to-end requirement because images may reveal identity or unrelated conditions even after direct identifiers are removed. Privacy claims should therefore specify the adversary and residual inference risk [254].

Consent and governance analyses add limitations that cryptography cannot solve. A technically secure system can still collect excessive data, reuse them outside the agreed purpose, or deny meaningful control to patients; these are design failures rather than paperwork issues [255]. XAI reviews distinguish feature attribution, saliency, example-based explanation, and interpretable models. These methods answer different questions and can be unstable, so an explanation should not be treated as proof that the model used medically valid reasoning [154]. Healthcare-5.0 analyses position explainability as part of trust and accountability. The appropriate explanation depends on the user: a developer needs failure diagnostics, a clinician needs evidence relevant to a decision, and a patient needs understandable consequences and alternatives [153].

Explainable medical-image analysis that combines learning with knowledge reasoning attempts to connect predictions to clinical concepts. This is more useful than an isolated heatmap when the knowledge base is valid and the reasoning chain is tested with clinicians [256]. Explainability in IoMT security allows operators to inspect why traffic was flagged. The explanation target is operational rather than diagnostic, and its utility should be measured by faster, more accurate incident response rather than visual plausibility [257].

A data-centric synthesis connects quality, security, privacy, and explainability because all depend on traceable data provenance. This integrated view is stronger than treating trust as a collection of add-ons after model training [240]. Table 13 summarizes representative threats and the mechanisms marshalled against them.

## 11. Diagnostic Datasets, Reference Standards, and Evaluation

The trust requirements above can be verified only through designs that expose failure rather than reward one headline score. Progress in diagnostic IoMT is bounded by acquisition quality, label validity, and benchmark design. A dataset should identify the clinical population, recruitment setting, device and protocol, timing relative to disease and treatment, and the process used to establish the reference standard. Without this information, the same numerical task can represent convenient case–control discrimination rather than the intended screening or diagnostic use.

Accuracy is misleading for rare events because an always-negative model may appear strong. Sensitivity, specificity, predictive values, likelihood ratios, calibration, confidence intervals, and the locked decision threshold are needed; continuous monitoring additionally requires false alarms per patient-time, detection latency, data coverage, and the proportion rejected by signal-quality or uncertainty gates. For prognostic tasks, the prediction horizon and censoring process must also be explicit. These metrics should be reported per patient and clinically meaningful subgroup, not only per window or image.

Cross-dataset stress detection directly demonstrates distribution shift: performance can fall sharply when devices, protocols, and populations change. Such a decrease is not an inconvenient secondary result but evidence about expected deployment behavior [217]. This observed shift motivates hierarchical domain adaptation as a response to the failure of within-subject evaluation. Adaptation performance should be reported separately for known and unseen domains and should include a mechanism for refusing predictions when the new domain is too different [66]. Federated-learning data surveys show that fragmented standards, scarce labels, and inconsistent definitions obstruct fair comparison. Federation can make more data participate, but it cannot repair incompatible labels or biased collection [130].

A maternal-health dataset collected through IoT-enabled cyber-physical systems across nine facilities is valuable because site diversity makes external-style evaluation possible. Its usefulness depends on preserving facility, patient, acquisition, and outcome provenance so models can be split at the correct unit [258]. The resulting rule is simple: report metrics that correspond to the clinical action, split data at the patient and deployment boundary, quantify uncertainty and missingness, and describe provenance sufficiently for the claim to be reproduced.

## 12. Cross-Cutting Diagnostic Evidence and Translation

The preceding metrics become meaningful only when they are matched to the maturity of the diagnostic claim. Most disagreements in intelligent IoMT are not about whether AI can classify data, but about what a reported result proves. A high score may establish separability within a dataset; a target-device experiment may establish compute feasibility; an independent-site study may establish partial transportability; and a prospective workflow study may establish diagnostic or clinical utility. These evidence levels must remain separate because success at an earlier level is necessary but does not imply success at the next.

### 12.1. Architecture Must Be Evaluated with the Model

Fog-computing experiments are strongest when they report prediction and service metrics together. The stochastic monitoring study that achieved 92% classification accuracy and 56% lower latency is informative because it exposes a joint performance–QoS trade-off. Its simulation setting also defines the limit of the claim: queueing and classification were tested, whereas physical-device failures and clinical response were not [42]. Embedded glucose prediction advances the evidence by combining three clinical datasets, a low-power wearable, a smartphone interface, cloud fine-tuning, and hardware-in-the-loop control trials. The study demonstrates that model architecture and compute placement can be evaluated as one system. Its remaining gap—in-silico intervention rather than prospective patient management—shows exactly where technical validation ends [52].

The lightweight cardiac framework adds another important dimension by reporting footprint, RAM, latency, explanations, attack probability, and external-dataset performance. Its AUC decline from MIT–BIH to PTB-XL and Chapman is not a weakness to hide; it quantifies the distribution-shift cost that a single internal score would miss [44]. In contrast, the federated wearable-and-implantable architecture reports favorable accuracy, latency, and communication cost using synthetic multimodal data. This is useful for controlled comparison of system components, but it cannot validate sensor noise, patient heterogeneity, or adversarial behavior in real devices. The correct conclusion is feasibility under simulation, not readiness for high-priority clinical deployment [81].

Together, these studies imply a reporting rule: every claimed architectural benefit should be paired with the model metric it changes, and every model result should state the hardware and data path on which it was obtained. Reporting only one side makes it impossible to determine whether an improvement survives the complete IoMT service.

### 12.2. Very High Accuracy Often Signals a Validation Question

Near-perfect results occur repeatedly in EEG and medical-image studies. A dynamic graph transformer reported approximately 99% on established emotion datasets, but affective labels, laboratory acquisition, and subject dependence limit clinical interpretation. The number is evidence about the benchmark task, not about mental-health diagnosis in natural settings [65]. The depression-monitoring framework reported 99.92% and 99.68% accuracy on two public datasets. Use of two datasets is stronger than a single internal split, yet the evidence remains retrospective and does not establish prospective diagnosis, monitoring stability, or performance with comorbidity. Extremely high values should prompt inspection of subject separation and shortcut cues rather than automatic acceptance or rejection [90].

Neonatal sleep staging provides a more informative validation pattern by combining k-fold and leave-one-subject analysis and reporting both accuracy and kappa. It also discloses cohort sizes of 64 and 19 neonates, allowing the reader to see that subject-level testing improves rigor while the smaller cohort still limits precision [86]. Cross-dataset stress analysis supplies the clearest evidence of why internal performance is insufficient. When performance changes across datasets, the decrease measures sensitivity to population, sensor, protocol, and label definition. A survey should treat this loss as a primary result because it estimates the cost of moving from development to deployment [217]. The resulting comparison argues against ranking models by headline accuracy. Evidence maturity is better described by the split unit, number of independent sites, temporal separation, target-device testing, calibration, and prospective use. Table 14 summarizes the evidence levels used to bound these claims.

### 12.3. Structured Appraisal of Representative Diagnostic Evidence

The six studies in Table 15 were selected purposively rather than statistically. Selection required that a study (i) contribute directly to a recurring conclusion about diagnostic validity or deployment, (ii) report enough information to appraise the relevant bias-sensitive domains, (iii) add variation in evidence maturity across retrospective validation, target-hardware testing, simulation, and synthetic experiments, and (iv) broaden coverage across physiological modalities, clinical tasks, and compute settings. Studies were not selected because they reported the highest performance. The set therefore illustrates how the appraisal framework changes interpretation across contrasting evidence classes.

These six studies are not intended to constitute a statistically representative sample and are not, by themselves, sufficient to support every conclusion in the main text. The paper’s conclusions derive from the complete thematic synthesis of 260 eligible evidence publications. Table 15 has the narrower purpose of making the evidentiary boundaries of selected recurring claims explicit. The appraisal is based on what each selected publication reports: an unreported safeguard is recorded as such rather than interpreted as present or absent, and “not applicable” is reserved for domains that cannot be meaningfully assessed in a purely synthetic experiment. The table is thus a structured interpretive safeguard rather than a numerical quality score.

### 12.4. From Discrimination to Operational Utility

Discrimination metrics answer whether positive examples tend to receive higher scores than negative examples; they do not determine whether a threshold is safe. For a continuous monitor, the threshold converts a probability into alerts and thereby determines missed events, false alarms, workload, and response time. A study should therefore report the threshold-selection procedure on validation data and lock it before testing. The chronic-disease fog–cloud comparison demonstrates why accuracy values around 95–99% cannot be interpreted without dataset and QoS context. Controlled benchmarks may be small, balanced, or curated, and performance can be inflated by leakage or overfitting. The review’s separation of diagnostic metrics from latency, jitter, throughput, bandwidth, and energy provides a more defensible comparison template [40].

Class imbalance makes precision strongly dependent on prevalence. An arrhythmia classifier evaluated on a balanced benchmark may produce many more false alerts when deployed in a mostly healthy population. Reporting sensitivity and specificity is not enough; precision should be estimated at an intended-use prevalence, and continuous systems should report alerts per patient-day. The end-to-end ECG monitoring study illustrates the value of class-specific results: KNN accuracy varied from 81% to 97% across arrhythmia and non-arrhythmia categories. An aggregate average would conceal the weakest classes, which may be the ones that most need escalation. Per-class confusion and clinical consequence are therefore more informative than a single headline number [91].

Calibration asks whether predicted probabilities correspond to observed risk. It matters when a probability sets the intensity of follow-up or when thresholds vary across clinics. A model can retain high AUC after a prevalence shift while becoming poorly calibrated, so discrimination and calibration must be evaluated separately. An evidential recurrent network for glucose prediction is relevant because it represents uncertainty while forecasting. Uncertainty becomes useful only when the workflow specifies what to do with it—for example, request another measurement, refer to a phone or cloud model, or alert a clinician. Otherwise it is an additional output without operational effect [52]. Latency should be measured from the beginning of the sensing window to receipt of an actionable output. Reporting only model inference excludes sampling duration, preprocessing, radio access, queueing, visualization, and human acknowledgment. The ESP32 cardiac framework’s 8.1-ms inference is strong device evidence, but it remains one component of the clinical response time [44].

Resource metrics should be similarly complete. Model parameters, file size, peak RAM, feature-extraction memory, average and worst-case latency, energy per inference, and update cost answer different questions. A compressed model may have a small file and still require large intermediate activations; a fast model may drain a battery through frequent sampling or communication. The TinyML ECG detector reports both 92.3% accuracy and 0.024-mW power, making its resource claim more interpretable than model size alone. The next evidence step is a long-duration device study that includes the sensor, preprocessing, radio, and false-alert workload under ordinary movement [83].

Clinical utility is reached only when the complete pathway is compared with current practice. The appropriate endpoint may be earlier treatment, reduced unnecessary testing, improved functional recovery, lower clinician workload, or safer care outside the hospital. The model metric remains an intermediate mechanism, not the final outcome.

### 12.5. Recurring Deployment Patterns

The surveyed systems converge on three deployment patterns. The first is a local screening cascade: a small model runs continuously, rejects clearly normal data, and escalates uncertain or suspicious windows to a stronger model or human reviewer. This pattern reduces communication and response delay while preserving a route for difficult cases. Multi-tier arrhythmia detection is an explicit example. AI modules are selected for heterogeneous nodes and inference is partitioned so emergencies trigger higher-level processing. Simulation supports compatibility and energy savings, while physical-node and patient testing remain necessary to establish timing and escalation reliability [82].

The second pattern is federated multi-site learning: institutions retain data and exchange protected updates. It is suitable when legal or organizational barriers prevent pooling and when sites have enough local observations to train. It is less attractive when clients are too small, labels are incompatible, or a secure centralized analysis is already permitted. Federated ECG decision-making across edge, fog, and cloud exemplifies the pattern’s promise. The architecture can combine diverse records without moving raw signals, but its success should be judged by per-site performance, communication rounds, straggler behavior, and update security rather than the global score alone [134].

The third pattern is a longitudinal patient model or digital twin. Sensor streams, records, and contextual data update an evolving state estimate that supports prediction or comparison of interventions. This pattern demands the strongest provenance because an incorrect identity, timestamp, or update can corrupt the patient’s trajectory. AI-generated patient and organ twins illustrate both the opportunity and the evidence burden. Historical trials, real-world records, wearables, and omics can support individualized prediction, but integration, trust, validation, and regulatory control remain unresolved. A twin should not be claimed merely because a dashboard updates in real time [60]. These patterns are not mutually exclusive. A wearable screening cascade can participate in federated training, and its outputs can update a longitudinal patient model. Combining them multiplies interfaces and failure modes, so each boundary must preserve patient identity, signal quality, model version, uncertainty, and authority to act.

Older ubiquitous-healthcare systems already combined edge and cloud for traffic prediction, caching, routing, and malicious-flow detection. Their proof-of-concept evaluations show the value of coordinated placement, but also illustrate that network optimization and clinical effectiveness require separate evidence [62]. Selection among the three patterns should begin with constraints. A seconds-critical alert favors local screening; a multi-hospital imaging model favors federation; and a slowly evolving chronic-disease trajectory may justify a longitudinal model. Adding a pattern without a corresponding constraint increases complexity without creating evidence.

The architecture should also include a degraded mode. Local screening needs behavior for sensor detachment and low battery; federation needs behavior when clients or the aggregator are unavailable; and a digital twin needs a validity indicator when observations are stale. Safe degradation is part of the intended function, not an exceptional maintenance state. Finally, all patterns require version control. The deployed artifact includes preprocessing, model parameters, threshold, feature definitions, software dependencies, and device configuration. Recording only a model filename is insufficient to reproduce which system generated a past clinical output.

### 12.6. A Minimum Reporting Contract

Weak evaluation is repeatedly identified alongside cloud dependence and fragmented security as a barrier to trustworthy edge healthcare. A minimum reporting contract makes studies easier to compare without forcing every application into one benchmark. The contract should follow the signal from patient and sensor through model and network to action [47]. At the cohort level, authors should report the clinical task, eligibility criteria, number of patients rather than only records, prevalence, site and time period, reference-label process, and the unit used for splitting. These fields reveal whether multiple segments, images, or visits from one person can leak across evaluation.

At the sensing level, device model, placement, sampling policy, calibration, missingness, artifact handling, and quality-rejection rules are required. Wearable cardiovascular evidence shows that field robustness is determined by these acquisition details, even when the downstream model is unchanged [76]. At the model level, preprocessing, architecture, tuning data, class-imbalance strategy, threshold selection, calibration, uncertainty handling, and ablation should be stated. The reproducible artifact is the whole pipeline, not only learned weights.

At the systems level, target hardware, peak memory, latency distribution, energy, communication volume, connectivity assumptions, security overhead, update mechanism, and degraded behavior should be measured. Communication-efficient edge-AI work shows that distributed intelligence creates costs that are invisible in model-only evaluation [53]. At the clinical-interface level, the recipient, displayed information, response deadline, confirmatory step, responsibility, and outcome of an alert should be specified. Critical-care loop frameworks are valuable precisely because they continue beyond prediction to monitoring, detection, correction, and follow-up [31]. At the trust level, the adversary, protected assets, authentication and authorization scheme, privacy parameters, explanation user, incident response, and audit trail should be explicit. A security metric cannot validate the diagnostic model, and a diagnostic metric cannot validate security. Table 16 consolidates these requirements into a minimum reporting contract.

### 12.7. Privacy, Security, and Explainability Are Testable Claims

Fog-based medical-text sanitization shows that privacy can be evaluated alongside utility, reporting 92% F1 for entity recognition and improved preservation of usable information. This is stronger than claiming privacy because data stayed near the source. It still does not quantify re-identification by an attacker, which must be tested separately [54]. Yet data locality alone is insufficient: generative inversion attacks show that exchanged representations can reconstruct private inputs. Privacy therefore depends on protocol messages and attacker access, not only on the location of the original database. A credible FL paper should test leakage or state explicitly that it has not done so [139].

Blockchain-secured FL reviews find that auditability and decentralized trust are common goals, but experimental metrics and threat models remain inconsistent. The technology should be justified by a failure of a simpler trusted aggregator, and its latency and storage overhead should be included in the comparison [147]. Modern intrusion-detection benchmarks expose a parallel issue: attack classes are imbalanced, so overall accuracy can hide failure on rare but dangerous threats. Per-attack recall and temporal or device-held-out testing are the security equivalents of sensitivity and external validation in clinical prediction [245]. Explainability is testable in the same way. An explanation can be evaluated for fidelity, stability, clinician usefulness, and effect on decisions. Without those tests, an attention map or generated rationale is an interface output rather than evidence that the model reasons clinically [154].

### 12.8. Clinical Value Depends on the Last Mile

A multi-facility maternal-health dataset demonstrates why provenance is a translation asset. Data collected across nine facilities can support facility-held-out evaluation, but only if site, patient, acquisition, and outcome identities are preserved. Removing these identifiers for convenience can make an apparently clean benchmark scientifically weaker [258]. Successful-ageing research shows that accessibility, privacy, interoperability, and adoption determine whether technically correct monitoring reaches users. These factors belong inside effectiveness evaluation because non-use and exclusion reduce real-world sensitivity even when model performance is unchanged [227].

Remote perioperative monitoring illustrates the final-mile metric problem. The clinically meaningful outputs are earlier escalation, fewer unplanned encounters, manageable alert burden, and safe follow-up. A system that detects physiologic deviations but does not change any of these outcomes remains a monitoring prototype [238]. The synthesis yields one central design rule: begin with the clinical action and work backward. Define who receives the output, how quickly it must arrive, what evidence confirms it, and what happens when data or confidence are inadequate. Sensor choice, model family, compute placement, privacy mechanism, and explanation interface should then be selected to satisfy that pathway rather than optimized independently.

## 13. Emerging Directions for Diagnostic IoMT

**Figure 9:**
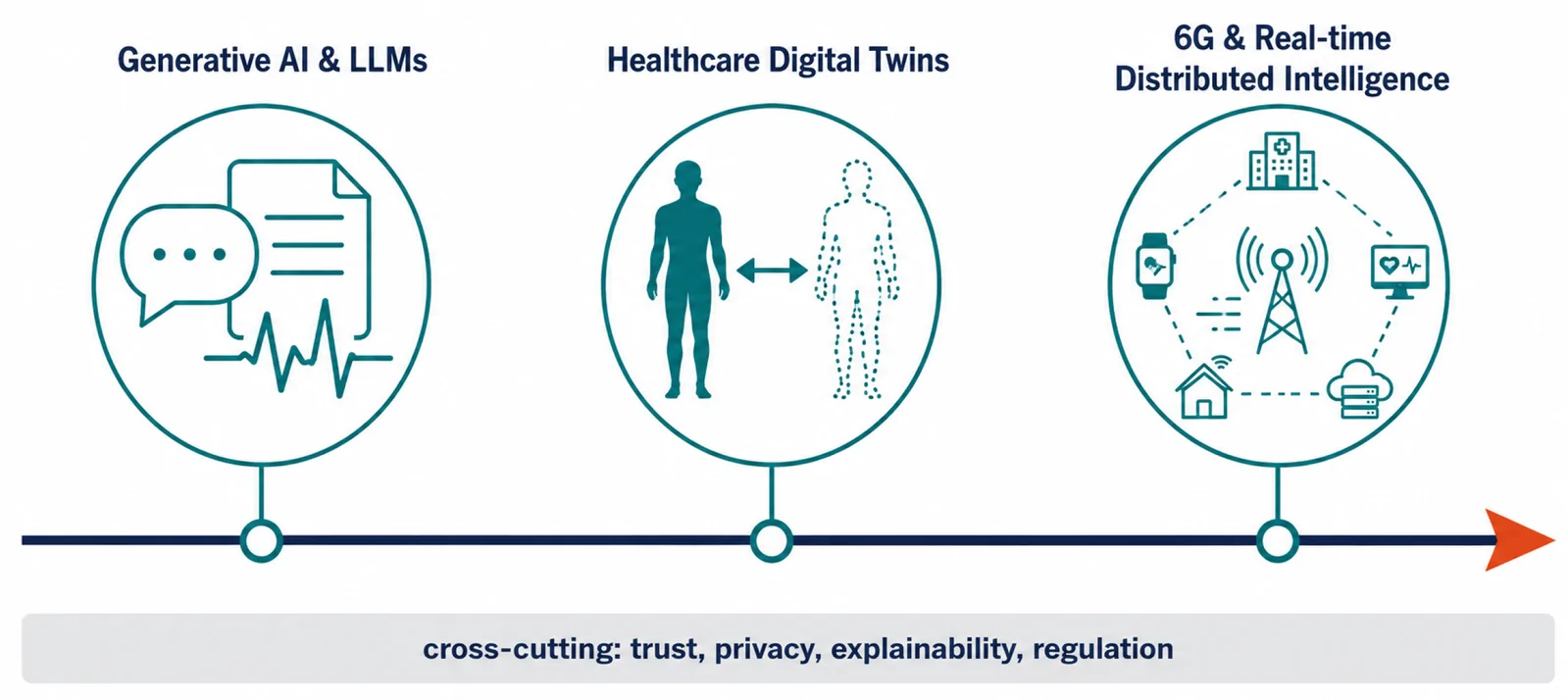
Emerging directions for diagnostic IoMT. Generative models, longitudinal patient representations, and next-generation connectivity can extend interpretation and access, but remain bounded by measurement validity, verification, privacy, and clinical governance. The arrows indicate proposed development pathways toward clinically governed deployment; they do not represent established causal relationships or guaranteed performance improvements.

With the evidence boundary established, emerging technologies can be judged by their contribution to a defined diagnostic pathway rather than by novelty alone. Generative interfaces may synthesize evidence, digital twins may represent longitudinal prognosis, and new materials may move analytical measurements closer to the patient. None replaces a reference standard, calibrated threshold, or clinician-governed escalation; Figure 9 sketches these bounded roles.

### 13.1. Evidence Boundary for Exploratory Infrastructures

Blockchain, quantum learning, next-generation connectivity, large language models, and digital twins are treated here as emerging directions unless the cited work includes target-device, external, prospective, or workflow evidence. Blockchain may support provenance but does not validate a diagnostic model; a quantum or 6G simulation does not establish hardware or clinical advantage; an LLM interface does not establish diagnostic benefit; and a synchronized dashboard is not a validated patient twin. Digital-twin-assisted quantum federation and quantum-blockchain reinforcement learning therefore remain conceptual or simulation-level architectures requiring matched classical baselines, realistic hardware and communication accounting, component ablation, and independent clinical validation before any diagnostic advantage can be claimed [259,260]. This boundary applies the same caution used for federated learning rather than assigning credibility from technological novelty.

### 13.2. Generative Interfaces for Diagnostic Evidence

Generative AI and large language models can summarize heterogeneous records and provide natural-language interfaces for diagnostic evidence, potentially extending access to specialist knowledge. Their fluency creates a distinctive risk: an unsupported synthesis may appear clinically authoritative. Evaluation must therefore include factual consistency with authorized source data, omission of critical abnormal findings, uncertainty communication, escalation, and local workflow fit rather than linguistic quality alone [261].

A retrieval-augmented multimodal LLM for IoMT data management grounds responses in authorized sources rather than model memory alone. Retrieval reduces but does not eliminate error; the system must verify source relevance, prevent cross-patient leakage, and expose citations and uncertainty to the user [262]. The appropriate near-term role is evidence retrieval, structured summarization, and communication support, not autonomous diagnosis. When generated text influences a diagnostic decision, the evaluated system includes retrieval, prompt and model version, source display, user verification, and the fallback when evidence conflicts.

Zero-trust context-aware access management is particularly relevant when generative systems can retrieve across cloud health resources. Every request should be authorized by user, patient, purpose, and context, and generated outputs should inherit the access restrictions of their sources [263].

### 13.3. Longitudinal Patient Models and Connected Biosensors

A neonatal-care framework combining IoMT streams and prediction illustrates a proposed patient twin in a vulnerable population. The diagnostic value of a twin depends on an explicit state definition, synchronized observations, uncertainty when data become stale, and prospective comparison with specialist assessment. A continuously updated dashboard is not a validated twin merely because it represents one patient [264].

Longitudinal patient models are most defensible when they estimate a time-defined risk or compare an observed trajectory with an expected one. Their provenance burden is unusually high: patient identity, time, treatment changes, sensor calibration, and model version must remain aligned. Counterfactual treatment claims require causal evidence beyond ordinary prediction, and the system must expose when missing or shifted data invalidate the state estimate [60].

New sensor materials can expand the diagnostic measurements available outside centralized laboratories. Intelligent hydrogels may support responsive wound assessment, while connected microfluidic and optical biosensors may support molecular or cellular detection. Their path to clinical use begins with biocompatibility, analytical sensitivity and specificity, interference testing, repeatable manufacturing, specimen handling, and agreement with established assays; network connectivity and AI become relevant only after the measurement itself is reliable [8,265].

## 14. Open Challenges and Future Directions

The emerging directions do not remove the field’s established diagnostic constraints; they make them more consequential. Data heterogeneity and label scarcity limit generalization because hospitals and devices do not observe identical populations or apply identical reference standards. Federation, self-supervision, and domain adaptation address parts of this problem, but none repairs a weak label, spectrum bias, or missing provenance [130].

Energy and compute limits cap persistent on-device intelligence. Compression, TinyML, and split computation move inference outward, but their evaluation must include target-hardware latency, memory, energy, and accuracy under the intended signal conditions [126]. Even after hardware constraints are met, distribution shift undermines models that look strong within one dataset. Personalization and continual learning can respond, but they also create moving software whose updates require monitoring, versioning, and revalidation [66].

Trust and accountability remain partially met. Explainable AI can support review, while prospective evaluation must establish whether explanations and predictions improve diagnostic decisions rather than merely appear plausible [153]. Privacy requires more than local data storage, and regulation requires more than encryption. Formal privacy, secure aggregation, identity, consent, purpose limitation, and incident response must be combined under a standardized governance process [255]. Interoperability remains a prerequisite because diagnostic models cannot safely consume data whose units, timing, patient identity, reference labels, and clinical semantics are inconsistent. The field therefore needs co-design of sensors, models, reference standards, interfaces, and confirmation pathways rather than a single algorithmic breakthrough [2]. Table 17 converts these gaps into diagnostic research priorities.

## 15. Limitations of the Survey

This survey has five limitations. First, literature identification used Scopus as the principal bibliographic database; relevant studies indexed only elsewhere may therefore be absent. Second, the English-language and 2018–14 August 2026 limits emphasize recent work but can exclude earlier or non-English evidence, except for selected standards retained to support technical interpretation.

Third, the integrative narrative design used iterative, purpose-specific searches and purposive synthesis rather than a fixed systematic-review cohort. Because the searches were not maintained as one auditable screening log, the exact numbers identified before deduplication and screened at title/abstract stage cannot be reconstructed reliably. The review should therefore not be interpreted as an exhaustive prevalence estimate, systematic-review denominator, or meta-analysis.

Fourth, the underlying literature is heterogeneous in task definitions, cohorts, devices, split units, metrics, and validation maturity. Numerical results are consequently interpreted within their own study design and are not pooled into a leaderboard. Fifth, some recent conference or online-first records provided less methodological detail than mature full-length clinical studies. When patient-level separation, reference-standard construction, calibration, or external validation could not be established from the reported material, the manuscript marks the evidence as limited or not reported rather than assuming the missing safeguard.

These limitations also bound the review’s conclusions. The literature supports technical feasibility for many sensing, learning, and distributed-computing components, but much of that support comes from retrospective benchmarks, target-device prototypes, simulations, or conceptual architectures. External retrospective validation provides stronger evidence of transportability, while prospective silent and workflow studies are required before a claimed diagnostic advantage or patient benefit is accepted. Recommendations in this survey are therefore framed as reporting and validation requirements, not as evidence that every technology discussed is clinically effective.

## 16. Conclusions

*Findings from the literature synthesis.* The Internet of Medical Things can extend diagnostic observation beyond the clinic, but measurement, detection, screening, diagnosis, prognosis, and decision support remain different claims. This survey followed diagnostic information from physiological, imaging, and molecular acquisition through quality control, edge–fog–cloud computation, machine and deep learning, federated collaboration, and clinician-governed action. Across modalities, the same conclusion emerged: diagnostic performance belongs to the complete pathway, not to an isolated model.

Architecture and learning choices matter because they change clinical error. Local inference can reduce delay while compression may alter sensitivity; privacy mechanisms can protect patients while changing calibration; and federation can broaden participation while leaving site-specific failure hidden by a global score. These trade-offs are defensible only when evaluated against the intended diagnostic role and target hardware. Likewise, a connected ECG classifier, imaging model, seizure detector, or biosensor should not be described as diagnostic without a suitable reference standard, patient-level data separation, locked thresholds, uncertainty and referral policies, external or temporal validation, and evidence that the output reaches an accountable workflow.

*Recommendations proposed by the authors.* The most productive direction is therefore not greater automation in the abstract, but stronger linkage between the clinical question and every technical choice. Monitoring systems should prove continuity and manageable alert burden; screening systems should prioritize sensitivity and confirmatory access; diagnostic systems should establish spectrum-appropriate accuracy and calibration; prognostic systems should define time and censoring; and decision-support systems should demonstrate beneficial human–AI interaction. Security, privacy, explainability, and lifecycle monitoring are parts of this diagnostic assurance case. Future IoMT research will create clinical value when it reports not only what a model predicts, but what was measured, against which reference, for whom, under what shift, with what uncertainty, and what safe action follows.

## Figures and Tables

**Table 1:** Diagnostic roles of AI-enabled IoMT outputs and the evidence each role requires.

Role	Question Answered	Required Evidence Emphasis	Typical Output
Monitoring	Has a measured state or trajectory changed?	Signal validity, uptime, missingness, drift, and alert burden	Trend or threshold event
Screening/early detection	Who should receive confirmatory assessment?	High sensitivity, prespecified threshold, representative spectrum, and referral pathway	Screen positive/negative
Diagnostic classification	Is a condition present, and of what type or severity?	Independent reference standard, sensitivity and specificity, calibration, subgroup and external validation	Probability or diagnostic category
Prognosis	What outcome is likely within a defined horizon?	Time origin, censoring, calibration over time, competing risks, and temporal validation	Individualized risk
Clinical decision support	What information or action should be considered next?	Human factors, uncertainty display, override and escalation behavior, and effect on decisions or outcomes	Prioritized review or recommendation

**Table 2:** Coverage of representative surveys compared with the present survey along six selected dimensions of the sensing-to-decision pipeline. The operational definitions and rating procedure are specified in Section 2; the ratings measure scope coverage rather than methodological quality.

Representative Survey (Focus)	Sensing/Wearables	Edge–Fog–Cloud	ML & DL Analytics	Federated Learning	Clinical Domains	Trust/Privacy
Ali et al. [11] (FL in healthcare)	Partial	Limited	Partial	Deep	Partial	Deep
Prasad et al. [12] (FL for IoMT)	Partial	Deep	Deep	Deep	Partial	Deep
Nithyavani et al. [13] (IoMT security)	Deep	Partial	Deep	Limited	Deep	Deep
Qureshi et al. [15] (AI & biosensors)	Deep	Partial	Deep	Partial	Deep	Partial
Karar et al. [16] (single application)	Deep	Partial	Deep	Limited	Deep (single domain)	Limited
Kakhi et al. [3] (AI and IoMT)	Deep	Partial	Deep	Limited	Partial	Partial
Messinis et al. [14] (AI for IoMT security)	Partial	Limited	Deep	Deep	Limited	Deep
Ashfaq et al. [17] (enabling technologies)	Deep	Partial	Partial	Limited	Partial	Partial
Al-Turjman et al. [18] (applications and trends)	Deep	Partial	Partial	Limited	Deep	Partial
Gallo and Micucci [19] (non-functional factors)	Limited	Deep	Limited	Limited	Limited	Deep
Matthew et al. [4] (state of the art)	Deep	Partial	Partial	Limited	Deep	Partial
Pinto et al. [20] (FL anomaly detection)	Limited	Limited	Deep	Deep	Limited	Deep
Ahmed et al. [21] (transformative impact)	Deep	Partial	Deep	Partial	Deep	Deep
**This survey**	**Deep **	**Deep**	**Deep**	**Deep**	**Deep**	**Deep**

**Table 3:** Engineering trade-offs interpreted through their diagnostic consequences.

Trade-Off	What Is at Stake	Primary Resolution Mechanisms	Main Section
Latency vs. accuracy	A smaller local model may escalate earlier but miss subtle cases or miscalibrate risk	Cascaded screening, uncertainty-based referral, split inference, and prespecified deadlines	Section 4, Section 6
Energy vs. privacy	Protection and local learning consume energy, while unprotected transmission exposes diagnostic data	Lightweight cryptography, secure aggregation, calibrated differential privacy, and duty cycling	Section 7, Section 10
Model size vs. domain shift	Compression may preserve average discrimination while degrading minority-class sensitivity across sites or sensors	Subgroup testing, domain adaptation, personalization, and post-compression calibration	Section 6, Section 7

**Table 4:** Evidence classes used to bound interpretation in this integrative survey.

Evidence Class	Directly Demonstrated	Interpretive Boundary
Prospective clinical/workflow study	Performance on live patients or data acquired prospectively in the intended pathway	Can support operational or clinical utility only for the tested setting and outcome
External or temporal retrospective validation	Locked analysis on a separate institution, device, population, or period	Supports partial transportability, but not prospective workflow benefit
Internal retrospective benchmark	Algorithm evaluated within one dataset or development environment	Supports discrimination under the stated split; leakage, calibration, and transportability remain concerns
Target-hardware prototype	Processing, memory, energy, or communication measured on intended or representative hardware	Supports engineering feasibility, not clinical accuracy or benefit by itself
Simulation/synthetic experiment	Controlled model, network, or synthetic-data experiment	Supports comparative behavior under assumptions; physical and clinical validity remain unproven
Conceptual, review, or emerging proposal	Taxonomy, architecture, requirements, or proposed future system	Supports problem framing and hypothesis generation, not an effectiveness claim

**Table 5:** Characteristics of the IoMT compute tiers and the intelligence typically deployed at each. Latency observations are indicative and workload dependent, not fixed architectural bounds.

Tier	Representative Devices	Indicative Latency Evidence	Compute/Energy	Intelligence Typically Deployed
Sensing/BAN	Wearables, implants, ECG patches	Sub-10-ms inference has been reported for an optimized ESP32 model [44]	Very low	Lightweight thresholds, tiny ML, feature extraction
Edge/gateway	Smartphone, home hub, embedded board	Milliseconds to hundreds of milliseconds in task-specific prototypes [44,45,46]	Low	Compressed DL inference, anomaly detection, BAN aggregation
Fog/local	Edge server, hospital LAN node	Network- and queue-dependent; simulation reports relative reductions rather than a universal bound [42]	Medium	Ward-level aggregation, model caching, partial training
Cloud/data center	Cloud GPU clusters	Path-dependent; transport, queueing, and service load must be measured end to end [39,40]	Very high	Large-model training, population analytics, durable storage

**Table 6:** Communication technologies commonly used across IoMT tiers and their typical roles.

Technology	Scale/Range	Data Rate	Power	Typical IoMT Role
BLE/Zigbee	Body area, <10–100 m	Low–medium	Very low	Sensor-to-gateway body-area links
Wi-Fi	Local, <100 m	High	Medium–high	Home/ward gateway uplink
LoRa/NB-IoT	Wide area, km-scale	Very low	Very low	Sparse remote monitoring, rural telehealth
4G/5G cellular	Wide area	High	Medium	Mobile monitoring, low-latency offload
6G (emerging)	Wide area	Very high	Medium	Real-time distributed intelligence, massive density

**Table 8:** Classical machine learning versus deep learning for IoMT analytics.

Dimension	Classical Machine Learning	Deep Learning
Input	Engineered features, tabular data	Raw signals, images, sequences
Data appetite	Effective with small datasets	Needs large labeled datasets
Compute/energy	Low; runs on sensing/edge tier	High; often needs edge/cloud accelerators
Interpretability	Comparatively high	Lower; needs explainability tools (Section 10)
Robustness to scarcity	Degrades gracefully	Sensitive; needs transfer/augmentation
Typical use	Screening, risk scores, tabular EHR	Imaging, ECG/EEG morphology, multimodal fusion
Typical IoMT placement	Sensing/edge tier	Edge/fog/cloud tier

**Table 9:** Model-compression techniques for deploying deep learning on constrained IoMT devices.

Technique	Mechanism	Primary Gain	Main Risk
Pruning	Remove redundant weights/channels	Smaller, faster model	Accuracy loss if over-pruned
Quantization	Reduce numerical precision (e.g., to int8)	Lower memory/energy	Precision-sensitive tasks degrade
Knowledge distillation	Small student mimics large teacher	Compact yet accurate	Requires good teacher, extra training
Neural architecture search	Automatically design compact models	Tailored efficiency	High search cost

**Table 10:** Federated learning strategies for the IoMT and the problem each primarily addresses.

Strategy	Mechanism	Primary Problem Addressed
Adaptive/weighted aggregation	Adjust how local updates are combined into the global model	Statistical heterogeneity across sites
Personalization/fine-tuning	Adapt the global model to each site or patient	Non-identical data distributions
Secure aggregation & differential privacy	Combine or perturb updates so individuals are protected	Information leakage from updates
Communication-efficient FL	Compress or reduce the frequency of update exchange	Bandwidth and intermittent connectivity
Client selection/asynchronous FL	Choose or tolerate stragglers among devices	Device (system) heterogeneity
Blockchain-assisted FL	Decentralize trust and audit the aggregation	Integrity, single-point-of-failure, provenance

**Table 11:** Diagnostic IoMT modalities, defensible roles, and evidence needed beyond retrospective model performance.

Modality	Example Target	Defensible Connected-AI Role	Critical Additional Evidence	Examples
Multilead ECG	Arrhythmia and multilabel cardiac findings	Screening, prioritization, and decision support	Patient/site-held-out sensitivity, calibration, lead loss, motion artifact, and device transfer	[155,156]
EEG/movement	Epileptic seizure or seizure phenotype	Event detection and escalation	Event-level sensitivity, false alarms per hour, latency, neurologist-adjudicated video EEG, and mimic spectrum	[157,158,159]
Radiography/CT	Lung abnormality, pneumonia, or organ delineation	Computer-aided detection, segmentation, and review prioritization	Protocol shift, external cohorts, pathology/follow-up reference, explanation fidelity, and reader study	[160,161,162]
Microscopy	Leukemia cell classification	Preliminary classification and remote prioritization	Slide and patient separation, staining/site shift, hematopathology reference, and full diagnostic-work-up concordance	[163]
Molecular/biosensor	Pathogen, fusion gene, antigen, exosome, or circulating cell	Decentralized screening or point-of-care testing	Analytical sensitivity/specificity, interference, lot and calibration effects, specimen workflow, and laboratory agreement	[7,8]
Longitudinal wearable data	Physiological deterioration or future event	Monitoring, early-warning, or prognosis	Time-defined outcome, missingness, alarm burden, temporal validation, and demonstrated escalation benefit	[76,165]

**Table 12:** Representative diagnostic and prognostic roles by clinical domain. The final column identifies the evidence that most directly limits translation.

Domain	Typical Sensing	Diagnostic Role	Confirmatory Reference	Key Translation Evidence
Cardiovascular	Wearable ECG, PPG	Rhythm screening, diagnostic prioritization, or prognosis	Standard ECG, expert interpretation, imaging, or adjudicated outcome	Event sensitivity, lead/device shift, calibration, and alert burden
Diabetes	Glucose sensor, urine or biochemical assay	Diabetes screening or glucose forecasting	Laboratory glucose/HbA1c or time-defined glucose outcome	Analytical agreement, temporal validation, and treatment-safe escalation
Oncology	Imaging, microscopy, and molecular biosensors	Lesion/cell detection, segmentation, or biomarker testing	Pathology, molecular assay, expert consensus, or follow-up	Spectrum and site shift, patient/slide separation, and concordance
Respiratory/infectious	Oximetry, audio, imaging, and pathogen biosensor	Deterioration screening or disease detection	Molecular assay, clinical adjudication, imaging follow-up, or outcome	Prevalence-aware predictive values, device robustness, and referral capacity
Neurological	EEG, movement, actigraphy, and speech	Seizure detection or neurological assessment	Video EEG, specialist assessment, or validated clinical instrument	False alarms per hour, detection delay, patient-held-out testing, and mimics
Older-adult care	Ambient and wearable sensors	Fall, frailty, or deterioration screening	Clinician/caregiver adjudication and subsequent outcome	Long-duration false alarms, adherence, accessibility, and response completion

**Table 13:** Representative trustworthiness concerns in the intelligent IoMT and common mechanisms.

Concern	Example Threat/Need	Common Mechanism
Channel security	Eavesdropping, spoofing, denial of service	Encryption, authentication, access control
Network intrusion	Attacks on IoMT devices/traffic	Learning-based intrusion detection (incl. GNN, ensembles)
Model security	Adversarial examples, data poisoning	Adversarial training, robust aggregation
Privacy	Re-identification, update leakage	Differential privacy, secure aggregation, data locality
Integrity/trust	Tampering, provenance	Blockchain audit trails, federated trust
Accountability	Unexplained decisions	Explainable AI (attribution, saliency, attention)

**Table 14:** Evidence levels for interpreting claims in intelligent IoMT studies.

Evidence Level	What Is Directly Tested	Defensible Claim	What Remains Unproven
Dataset experiment	Model on a retrospective benchmark	Algorithmic discrimination under the stated split	Device feasibility, external validity, clinical utility
Target-device test	Inference on intended hardware	Memory, energy, and latency feasibility	Population transportability and workflow benefit
Independent-site test	Locked model on another site/device	Partial robustness to distribution shift	Prospective behavior and effect on care
Prospective silent study	Live data without influencing decisions	Real-time data quality and operational performance	Human–AI interaction and patient benefit
Clinical utility study	Model integrated into the care pathway	Effect on decisions, workload, safety, or outcomes	Long-term drift and lifecycle performance
Monitored deployment	Versioned system under ongoing surveillance	Sustained service performance within scope	Generalization beyond the monitored context

**Table 15:** Bias-sensitive appraisal of purposively selected illustrative studies used in the main synthesis.

Study	Evidence Class	Patient-Level Separation	Reference Standard/Label Source	External or Temporal Validation	Calibration/Locked Threshold
Zhu et al. [52]	Retrospective longitudinal prediction + hardware-in-the-loop simulation	Personalized models; chronological train/validation/test separation within each subject	CGM measurements; hypoglycemia defined by three consecutive values below 70 mg/dL	Three clinical datasets; temporal holdout within subjects; no prospective patient-management test	Personalized uncertainty lower bound tuned on validation data; 70 mg/dL clinical threshold fixed
Cardiac ESP32 framework [44]	Target-device prototype + zero-shot cross-dataset test	Training and test sets reported; patient-disjoint construction of the MIT–BIH split not specified	Public ECG repository labels	Model trained on MIT–BIH and tested zero-shot on PTB-XL and Chapman	Brier score reported for the IDS; clinical ECG probability calibration and locked operating threshold not reported
Neonatal sleep staging [86]	Internal retrospective benchmark	Five-fold and leave-one-subject-out analyses reported	Sleep stages manually classified by expert neonatologists and used as the reference standard	Two cohorts from the same hospital; no independent-site or prospective validation	Probability calibration and a locked diagnostic threshold not reported
Cross-dataset stress analysis [217]	Cross-dataset retrospective validation	Subject-disjoint LOSO; all windows from one subject kept in the same fold	Laboratory protocol validated by STAI-S and cortisol; ambulatory labels derived from DASS-21 self-reports	Trained on MMSD and tested on the independently collected UWS dataset	Probability calibration and a prespecified operating threshold not reported
Fog stochastic study [42]	Queueing simulation + internal retrospective benchmark	Ten-fold CV on 303 Cleveland records; fold construction not further detailed	Cleveland heart-disease binary target; queue arrivals and service classes simulated	No independent clinical dataset or physical-site validation	No diagnostic probability calibration; 1.2-ms SLA is an operational latency threshold
Federated wearable architecture [81]	Synthetic multimodal simulation	Non-IID partitions represented patients; no real-patient separation	Synthetic ECG, glucose, temperature, and SpO_2_ labels; morphology compared with public benchmarks	Statistical similarity checks only; no real-device or clinical external validation	Probability calibration and a locked diagnostic threshold not reported

**Table 16:** Minimum reporting fields for an evidence-centered intelligent IoMT study. Each requirement is linked to a recurring methodological failure and representative evidence in the reviewed literature.

Layer	Minimum Fields	Recurring Failure Addressed	Representative Evidence
Cohort and label	Patients, prevalence, sites, dates, eligibility, reference standard, split unit	Patient or segment leakage; spectrum and label bias	[65,86,90,217]
Sensor and signal	Device, placement, rate, calibration, artifacts, missingness, quality rejection	Hidden acquisition and device shift	[63,76,80]
Model and threshold	Pipeline, tuning, imbalance, calibration, threshold, uncertainty, ablation	Irreproducible tuning, forced prediction, and uncalibrated decisions	[52,91,217]
Compute and network	Hardware, memory, tail latency, energy, bytes, outage behavior, update cost	Deployability inferred from model-only evidence	[42,44,52,53,81]
Clinical workflow	Recipient, deadline, escalation, confirmation, responsibility, outcome	Alert produced without accountable action or utility evidence	[31,238]
Trust and lifecycle	Threat model, privacy parameters, access, explanations, versions, drift, rollback	Security, privacy, or explanation claimed without operational testing	[54,139,147,154]

**Table 17:** Open challenges and diagnostic research priorities for the IoMT.

Challenge	Promising Direction
Statistical heterogeneity and weak labels	Multi-site reference-standard harmonization; patient/site-held-out evaluation; robust domain adaptation
Device energy and compute limits	Model compression, TinyML, and split/edge computation
Distribution shift across sites and sensors	Locked external and temporal validation; monitored recalibration; safe abstention
Clinical trust and accountability	Explanation-user studies; uncertainty-based referral; prospective diagnostic workflow evaluation
Privacy and regulatory compliance	Privacy-preserving learning; standardized governance
Interoperability and integration	Standardized units, timing, provenance, reference labels, and diagnostic reports
Reliable generative AI	Source-grounded synthesis; omission and contradiction testing; clinician verification

## Data Availability

No new data were created or analyzed in this study. Data sharing is not applicable to this article.
